# Multicellular spatial model of RNA virus replication and interferon responses reveals factors controlling plaque growth dynamics

**DOI:** 10.1371/journal.pcbi.1008874

**Published:** 2021-10-25

**Authors:** Josua O. Aponte-Serrano, Jordan J. A. Weaver, T. J. Sego, James A. Glazier, Jason E. Shoemaker

**Affiliations:** 1 Department of Intelligent Systems Engineering, Indiana University, Bloomington, Indiana, United States of America; 2 Biocomplexity Institute, Indiana University, Bloomington, Indiana, United States of America; 3 Department of Chemical & Petroleum Engineering, University of Pittsburgh, Pittsburgh, Pennsylvania, United States of America; 4 McGowan Institute for Regenerative Medicine, University of Pittsburgh, Pittsburgh, Pennsylvania, United States of America; 5 Department of Computational and Systems Biology, University of Pittsburgh, Pittsburgh, Pennsylvania, United States of America; Temple University, UNITED STATES

## Abstract

Respiratory viruses present major public health challenges, as evidenced by the 1918 Spanish Flu, the 1957 H2N2, 1968 H3N2, and 2009 H1N1 influenza pandemics, and the ongoing severe acute respiratory syndrome coronavirus 2 (SARS-CoV-2) pandemic. Severe RNA virus respiratory infections often correlate with high viral load and excessive inflammation. Understanding the dynamics of the innate immune response and its manifestations at the cell and tissue levels is vital to understanding the mechanisms of immunopathology and to developing strain-independent treatments. Here, we present a novel spatialized multicellular computational model of RNA virus infection and the type-I interferon-mediated antiviral response that it induces within lung epithelial cells. The model is built using the CompuCell3D multicellular simulation environment and is parameterized using data from influenza virus-infected cell cultures. Consistent with experimental observations, it exhibits either linear radial growth of viral plaques or arrested plaque growth depending on the local concentration of type I interferons. The model suggests that modifying the activity of signaling molecules in the JAK/STAT pathway or altering the ratio of the diffusion lengths of interferon and virus in the cell culture could lead to plaque growth arrest. The dependence of plaque growth arrest on diffusion lengths highlights the importance of developing validated spatial models of cytokine signaling and the need for *in vitro* measurement of these diffusion coefficients. Sensitivity analyses under conditions leading to continuous or arrested plaque growth found that plaque growth is more sensitive to variations of most parameters and more likely to have identifiable model parameters when conditions lead to plaque arrest. This result suggests that cytokine assay measurements may be most informative under conditions leading to arrested plaque growth. The model is easy to extend to include SARS-CoV-2-specific mechanisms or to use as a component in models linking epithelial cell signaling to systemic immune models.

## Author summary

Respiratory lung infections form lesions in the lungs, whose number and size correlate with the severity of illness. In some severe cases, respiratory disease triggers a severe inflammatory condition known as a cytokine storm. In order to link molecular signaling at the site of infection to its impact on the overall interferon response and the occurrence of severe inflammation, we created a computational model of the early stages of infection that simulates lung cells infected with RNA viruses, such as those responsible for COVID-19 and flu, to help explore how the disease forms viral plaques, an *in vitro* analog to lesion growth in the lung. Consistent with experimental observations, our model suggests that the treatment of cells with type-I interferons, which are currently being evaluated for the treatment of COVID-19, may have a protective effect. We found that enhancing certain aspects of the inflammatory response, such as the JAK/STAT pathway, may be able to arrest viral plaque growth, suggesting molecules involved in this pathway as possible drug candidates. Analysis of the model also shows that to better estimate the parameter values needed to model interferon signaling and viral replication, experiments should be performed under conditions that inhibit viral growth, e.g., by pretreating cells with interferon. We present a computational framework built using the CompuCell3D multicellular simulation environment that lays the groundwork for constructing larger models of respiratory-infection-induced immune responses and that can inform experiments to improve our fundamental understanding of the mechanisms regulating the immune response.

## Introduction

Respiratory virus infections cause many deaths each year. Typical seasonal influenza virus strains are responsible for 290,000–650,000 annual deaths globally [1], and occasional, highly pathogenic pandemic strains, such as the 1918 Spanish Flu [2], and the 1957 H2N2 [3], 1968 H3N2 [4], and 2009 H1N1 [5] influenzas result in significantly higher mortality rates. As of June 29^th^, 2021, the SARS-CoV-2 virus, which causes COVID-19, has caused over 180 million recorded infections and 3.9 million deaths worldwide [6]. Both influenza and SARS-CoV-2 are RNA viruses, and studies of severe SARS-CoV-2 and influenza infections find that impaired interferon responses correlate with more severe outcomes [7–9]. In highly pathogenic infections, an aberrant inflammatory response–specifically a prolonged, elevated inflammatory state and a high level of type-I interferons in the bloodstream, clinically called hypercytokinemia (colloquially known as a cytokine storm) [10]–is believed to be a significant driver of mortality [11,12]. Excessive inflammation exacerbates tissue damage and hinders clinical recovery [13,14].

Influenza studies show that immunomodulation can improve infection outcomes. Prestimulation of toll-like receptors to induce earlier interferon production protects against highly pathogenic influenza strains in mice [15], while cell culture prestimulation with type-I interferons prevents viral plaque growth by SARS-CoV (the original 2003 SARS virus) [16], SARS-CoV-2 [16], and influenza [17]. Nebulized interferon α2b and interferon β are being investigated as an early treatment for COVID-19 [18,19]. Collectively, these studies demonstrate that immune response regulation must balance tissue damage from inflammatory responses against efficient viral clearance. Computational modeling may reveal how complex responses emerge during infection and aid in identifying immune-targeted treatments.

Recent computational models have considered many aspects of inflammatory responses to viral infection [20–23]. Ordinary-differential-equation (ODE) based models assume either homogeneity or a compartment-based quasi-spatial structure and typically ignore the diffusion of virus, local cytokine signaling, heterogeneity of cell responses to stimuli, and stochasticity of individual cells’ responses [20,24]. Recent models [21,25,26] of interferon response to viral infection commonly invoke a generic virally resistant cell type. A cell of this type is either immune to viral infection or stops ongoing viral replication completely. This all-or-nothing response does not capture the dynamics of interferon stimulated genes’ (ISGs’) effects on viral growth [25,26] or the nuances of partially resistant cells. Plaques are visible areas of infected and dead cells that occur in cell cultures infected with a virus. A spatial model of influenza viral spread and plaque growth [24] replicates the linear growth of viral plaques *in vitro* and explores the impact of diffusion coefficients on viral plaque formation but did not incorporate the cells’ interferon signaling response to the infection. Recent studies of DNA virus infection (Herpes simplex virus 2; HSV-2) used agent-based models to examine the role of adaptive immune cells in restricting plaque growth [27] while another study found that the degree of stochastic signaling minimized the amount of interferon needed to restrict cell death [28]. However, HSV-2 is a DNA virus that activates different signaling pathways from RNA viruses, such as influenza, and, as described above, severe respiratory infection often involves strong inflammatory signaling responses. This paper extends these approaches to explain plaque growth arrest due to ISGs for respiratory viral infections.

Plaque growth assays seed the virus at low multiplicity of infection (MOI) and allow it to replicate and form plaques across a monolayer of host cells in cell culture. We developed a multicellular spatial interferon signaling model (which we will call the MSIS model) of the early inflammatory response to RNA viral respiratory infections *in vitro* using CompuCell3D [29] (CC3D). MSIS simulations can replicate observed plaque growth, cytokine response, and plaque arrest. The MSIS model allows us to determine conditions that lead to either arrested or persistent plaque growth during a simulated infection of a monolayer of lung epithelial cells with an RNA virus. Plaque growth assays are commonly used to compare virus growth rates across cell lines [24,30], to quantify the concentration of infective agents [31,32], and to observe the effects of drugs and compounds on virus spread [33–36]. Simulation of *in vitro* experiments *in silico* allows for cheaper, faster, higher-throughput hypothesis generation than experiments. The MSIS model replicates familiar biological plaque growth assays and cell staining experiments, making its simulation methodology and results readily accessible to wet-lab biologists.

Our model focuses on two interacting processes: viral replication and the host cells’ early interferon response. The modeled virus is produced in infected cells, released into the extracellular environment, and diffuses in this environment. The modeled inflammatory response includes interferon production, export, diffusion and decay, and the induction of virally resistant cell states via ISGs. The model represents a monolayer of immobile human bronchial epithelial cells (HBECs). Each cell contains a separate model of epithelial cell interferon signaling, viral replication and release, and cell death, which is an ODE model [37] calibrated to data from influenza-infected HBECs, that has been modified to include species release or export to the extracellular environment. We adapted a standard model of cell types during viral infection [38], with cells transitioning from uninfected, to eclipse phase, virus releasing, and dead cell types. The extracellular environment allows for diffusion of both virus, which leads to the formation of viral plaques, and type-I interferons, which are responsible for paracrine interferon signaling. The MSIS model gives insight into the mechanisms of IFN regulation and the arrest of viral plaques.

## Materials and methods

The MSIS model simulates the replication and spread of an RNA virus infection in a monolayer of epithelial cells and the interferon response induced by the infection. Using CompuCell3D, we created simulations of the MSIS model that represent a diffusive extracellular environment above a square grid of discrete cells, each of which incorporates an ODE representation of epithelial cell interferon production in response to infection by an RNA virus.

### Spatial considerations of the MSIS model

During virus infection, lung epithelial cells produce and export virus and anti-viral type-1 interferon (IFN) proteins. In cell culture, these extracellular species diffuse freely in the medium above the apical surface of cells.

The conceptual model is that the apical surface of the epithelium interacts with the bottom surface of the medium in which extracellular IFN (IFN_e_) and virus (V_e_) diffuse and decay. We represent the cells and the chemical species in the extracellular medium as a cell lattice next to two chemical field lattices, one for IFN_e_ and one for V_e_. Cells export IFN and release virus from their apical surface into the adjacent domain in the chemical field. The CompuCell3D model is a 2D lattice model with the side of each voxel representing 3.0 microns. Unless otherwise specified, the simulation domain is a 300 by 300 lattice, representing a tissue patch of 900 by 900 μm. We represented the layer of epithelial cells using a 100 by 100 array of square cells, each occupying 3 by 3 voxel sites. The cells are infected by V_e_ and respond to IFN_e_ in the same adjacent domain. The area of the cell in the cell lattice represents the interface between the extracellular space and the cell’s apical surface.

Due to the spatial aspect of the model, the concentrations of extracellular species (IFN_e_ and V_e_) can be reported at specific lattice sites, averaged over the area of a cell, or averaged over the enter lattice. V_e_ and IFN_e_ indicate the concentration at a specific lattice site while [V_e_]_*per cell*_ and [IFN_e_]_*per cell*_ indicate the average concentration over a specific cell for extracellular virus and extracellular IFN, respectively. The model assumes no spatial variability within the cell.

### Cell types and rationale

During an RNA virus infection in lung epithelial cells, cells go through four distinct stages. Lung epithelial cells are interferon-competent and produce interferon in response to infection by a virus. During an infection, both infected and healthy cells are capable of responding to changes in extracellular IFN [39] via the JAK/STAT pathway. After infection, cells enter an eclipse phase for about 6 hours, during which they produce, but do not release, virus [40,41]. After the eclipse phase, cells begin to release virus and continue to do so until the cell’s resources are depleted, resulting in death.

**Fig 1:**
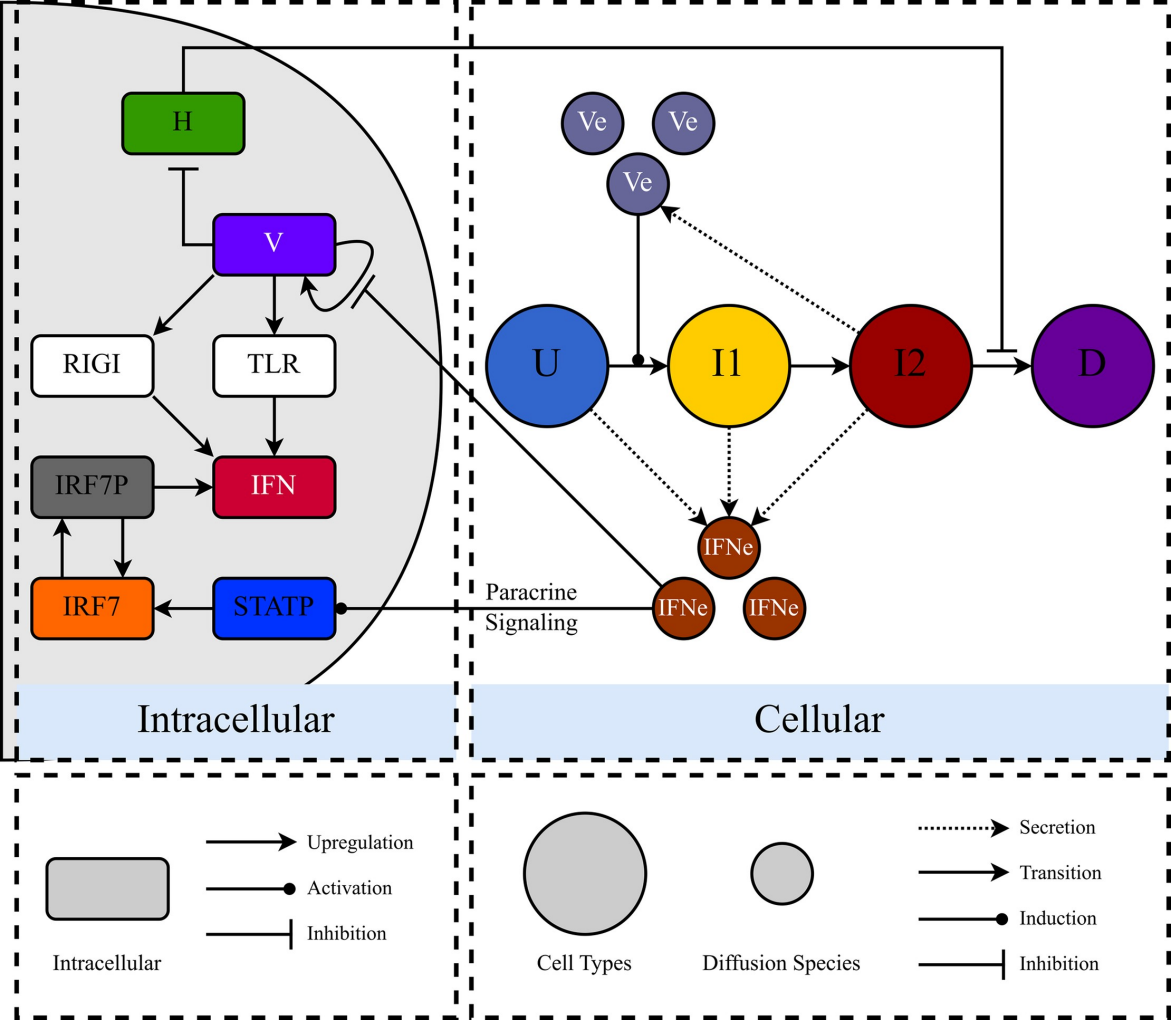
Conceptual diagram of the MSIS model. The MSIS model consists of an Intracellular sub-model, which describes intracellular interferon signaling during infection, and a Cellular sub-model, which defines changes in cell types and extracellular molecular diffusion. Uninfected cells (U, blue) produce IFN via paracrine signaling alone since no virus is present in these cells. Eclipse-phase cells (I1, yellow) produce IFN via viral sensor proteins (RIG-I and TLR7) and paracrine signaling (through the STAT pathway). I1 cells also export IFN into the extracellular environment. I1 cells allow virus replication but do not release virus into the extracellular environment. Virus-releasing cells (I2, red) produce IFN in a manner identical to I1 cells and export IFN and release virus into the extracellular environment. Dead (D, purple) cells do not interact with their surroundings and have no Intracellular sub-model. Cell type and chemical species colors are conserved throughout this work. Each cell contains an instance of the Intracellular sub-model representing interferon signaling (variables RIG-I, TLR, IFN, IRF7, IRF7P, and STATP), viral infection, replication, and release (V), and cell health (H). Type-I interferons (IFN_e_) exported by U, I1, and I2 cells, and virus (V_e_) released by I2 cells diffuse and decay in the extracellular environment. Paracrine interferon signaling occurs through the JAK/STAT pathway, indicated by the arrow from IFN_e_ to STATP across the Intracellular/Cellular border.

To model the four stages of infection a cell experiences, the CC3D-based MSIS model has cells (agents) with 4 distinct types: uninfected (U), eclipse phase (I1), virus releasing (I2), and dead cells (D). Fig 1 provides a conceptual overview of the MSIS model. Uninfected cells, U, contain no virus but can produce and export IFN in response to extracellular IFN via the STAT pathway. Paracrine signaling occurs when interferon external to the cell induces the phosphorylation of STAT (STATP in Fig 1). U cells transition to the eclipse phase (I1) immediately after a successful infection event. Eclipse-phase (I1) cells can produce and export IFN, and replicate, but not release virus [42]. Extracellular IFN (via paracrine signaling activation of the JAK/STAT pathway) and viral sensor protein (RIGI and TLR7) activation both stimulate cells to produce and export IFN. When an I1 cell transitions to the virus-releasing type (I2), all properties of the cell remain the same except that the cell can now release the intracellular virus into the extracellular virus field. When an I2 cell transitions to dead (D), it ceases to produce and export IFN or release virus but continues to occupy space in the simulation.

### Cell type transition probabilities

Transitions between cell types are stochastic, and the probability of a transition occurring depends on the simulation time step (Δ*t* = 10 minutes), the cell’s local extracellular and intracellular concentration of virus (V_e_ and V respectively), the cell’s health (H; described under Intracellular Model Equations and Rationale), and a transition rate coefficient (*β*, *k* or *γ*_*H*,*V*_). [V_e_]_*per cell*_ is measured as the local extracellular virus concentration each cell is exposed to over its entire cell area. We derived the rates in Eq 1 – 3 from the rate laws in [38], following the transformation rules given in [24]. When a cell is infected (transitions from U to I1), the internal viral concentration changes from 0 to 6.9E-8 (unitless), equivalent to a single virus particle entering the cell [37]. This amount of virus was considered negligible compared to V_e_ and is thus not removed from the extracellular virus. Within CC3D, cell-type transitions are implemented by sampling a random number for each cell between 0 and 1, inclusive, at each time step. The cell’s transition occurs when the probability, *P*, is greater than the random number. Each transition must occur in order.

Cell-Type Transition Probabilities

$$P(U\to I1)=1-exp(-\beta {\left[V_{e}\right]}_{per\;cell}\;\Delta t),$$ (1)

$$P(I1\to I2)=1-exp(-\tau_{I1}\Delta t)$$ (2)

$$P(I2\to D)=1-exp(-\gamma_{H,V}[V]\{1-H\}\Delta t)$$ (3)

### Intracellular model equations and rationale

Fig 1‘s “Intracellular” panel shows key molecules, species, and processes involved in an epithelial cell sensing RNA virus infection and producing IFN to suppress virus replication. During RNA virus infection of lung epithelial cells, the first immune system action is type I interferon production (IFNα and IFNβ), which suppresses virus replication and activates many downstream components of the innate and adaptive immune systems [9,43,44]. Interferons establish an antiviral state through the activity of interferon stimulated genes (ISGs), which include antiviral Mx proteins, RNA-activated protein kinases, and the 2 – 5A system [45]. The presence of an RNA virus is primarily sensed in the cytoplasm by retinoic acid-inducible gene 1 (RIG-I) and in endosomes by Toll-like receptors 7 and 9 (TLR7/9) [46,47]. Both RIG-I and TLR7/9 bind viral RNA that has been exposed by the uncoating of the virus. The activation of either sensor leads to the phosphorylation of interferon regulatory factor 7 (IRF7) and the production of interferons (see [39] for a detailed map of the relevant molecular interactions). Interferons are also auto-inducing. IFN excreted from the cell can bind interferon receptors (IFNR), which in turn activate the JAK/STAT pathway, leading to the production of more interferon. This positive feedback allows infected cells to produce a more robust interferon response (autocrine effect) while also priming an antiviral state in neighboring uninfected cells (paracrine effect). In summary, epithelial cells rely on interferon signaling to rapidly establish an antiviral state, and interferons are regulated through several pathways to ensure robust virus detection and response.

Another process that impacts virus reproduction is cell health. Studies have shown that the amount of virus produced by an infected cell decreases dramatically as the cell’s health decreases (quantified by its concentration of ATP [48]). Thus, even minimal models that seek to predict virus and interferon production dynamics should represent virus replication, interferon signaling dynamics, and the cell’s health.

The MSIS model adapts and extends to single cells the Weaver model of the dynamics of virus replication, interferon signaling, and cell health. The adapted Weaver model includes six ODEs (Eq 4 – 9) that define the rate equations for intracellular virus replication, interferon signaling, and cell health. Specifically, the equations define changes in the intracellular concentrations of virus (V [unitless]), interferon (IFN [μM]), phosphorylated STAT (STATP [μM]), IRF7 protein (IRF7 [μM]), and phosphorylated IRF7 (IRF7P [μM]). Eq 9 defines the dynamics of the health of the cell (H [unitless]). [IFN_e_]_*per cell*_ is the average extracellular interferon concentration each cell is exposed to over its entire cell area.

Rate equations for intracellular species and health adapted from Weaver *et al*:

$$\frac{d[V]}{dt}=\frac{k_{V,V}[H][V]}{1+\frac{{\left[IFN_{e}\right]}_{per\;cell}}{K_{V,IFNe}}}-Q_{V}[V]$$ (4)

$$\frac{d[IFN]}{dt}=[H](k_{IFN,V(RIGI)}V+\frac{k_{IFN,V(TLR)}V^{n}}{K_{IFN,V(TLR)}+V^{n}}+k_{IFN,IRF7P}[IRF7P])-Q_{IFN}[IFN]$$ (5)

$$\frac{d[STATP]}{dt}=\frac{k_{STATP,IFNe}[H]{\left[IFN_{e}\right]}_{per\;cell}}{K_{STATP,IFNe}+{\left[IFN_{e}\right]}_{per\;cell}}-\tau_{STATP}[STATP]$$ (6)

$$\frac{d[IRF7]}{dt}=[H](k_{IRF7,STATP}[STATP]+k_{IRF7,IRF7P}[IRF7P])-\tau_{IRF7}[IRF7]$$ (7)

$$\frac{d[IRF7P]}{dt}=k_{IRF7P,IRF7}[H][IRF7]-\tau_{IRF7P}[IRF7P]$$ (8)

$$\frac{d[H]}{dt}=-\gamma_{H,V}[H][V]$$ (9)

Below, we provide a brief description of the Weaver model and then discuss how we modified the model to support its implementation in the MSIS model (see Fig 1‘s Intracellular panel). A thorough description of the rate equations is available in [37].

The Weaver model groups interferon α and β into a single representative species, interferon (IFN). We modeled the inhibition of virus production in response to the cell’s spatially averaged level of extracellular interferon ([IFN_e_]_*per cell*_) using non-competitive inhibition-like kinetics (Eq 4). We used mass-action kinetics to describe the induction of IFN (Eq 5) by virus (via the RIG-I pathway) and IRF7P, and Hill kinetics to define the effect of the concentration of virus on IFN production via the TLR pathway. The rate of export of intracellular IFN into the extracellular environment obeys the concentration of IFN times a rate constant, *Q*_*IFN*_. We model extracellular IFN’s ([IFN_e_]_*per cell*_) activation of STAP with Michaelis–Menten kinetics (Eq 6), and mass-action kinetics are used to model the effect of STATP and IRF7P on the rate of production of IRF7 (Eq 7). We also use mass-action kinetics to describe the rate of IRF7P production as a function of IRF7 (Eq 8). In all equations, production terms are multiplied by the cell’s health (H) to represent the loss of production capacity in an infected cell. Heath is a relative metric bounded between 0 and 1, and the rate of the decay of health (Eq 9) is proportional to the concentration of virus in the cell and the health of the cell. All these rate laws are consistent with the original Weaver model.

We made three changes to the Weaver model to employ it in the MSIS model. We reinterpreted the first-order virus degradation term in the original Weaver model to represent the release of virus into the extracellular environment in the MSIS model. This term appears in Eq 4, where the rate of release of virus to the extracellular environment is proportional to the concentration of virus times a rate constant, *Q*_*v*_. The Weaver model was a population model, while Eq 4 – 9 represent the intracellular regulation of a single cell. The Weaver model has a state, P, which represents the fraction of live cells in the population. The mathematical equation for health is unchanged from the original Weaver model, but we have reinterpreted P to represent the health (H) of each cell. All production terms in Eq 4 – 8 are multiplied by the cell’s health (bound between 0 and 1) to represent the diminished production capacity of unhealthy, virus-infected cells. And, lastly, due to the spatial aspect of the MSIS model, we redefined the concentration of extracellular IFN in Eq 4 and 6 to be the average IFN_e_ over the area of a given cell; namely [IFN_e_]_*per cell*_.

In the multicellular spatial MSIS model, each live cell (U, I1, I2 types) has a replica of the rate equations (Eqs 4–9). Table 1 gives the initial conditions for each cell type. Table 2 lists the parameters for the rate equations. For U and I2 cell types, the equations and their parameter values are unaltered. In I1 cells, the equations are the same and all parameter values are unchanged except for the parameter value that defines the rate of virus release into the extracellular environment, *Q*_*v*_, which is set to zero because eclipse phase cells (I1) do not release virus. All parameter values are listed in Table 2. Now that the mathematics of each cell’s dynamic responses have been defined, the spatial considerations must be accounted for when determining the concentrations of extracellular IFN (IFN_e_) and extracellular virus (V_e_) on a per-cell basis.

**Table 1:** Initial conditions for each cell type when present at the start of a simulated infection.

		Cell Type			Units
		U	I1	I2	
Intracellular Species Initial Value	IFN	0	0	0	μM
	STATP	0	0	0	
	IRF7	0	0	0	
	IRF7P	0	0	0	
	H	1	1	1	unitless
	V	0	6.90E-8	6.90E-8	

**Table 2:** Baseline parameter values and sources. Parameters marked with * are specific to the CompuCell3D’s implementation of the simulation.

Parameter	Value	Units	Process	Equation(s)	Source
*k*_*IFN*,*V(RIGI)*_	0.0	μM hr^-1^	Rate of IFN production via RIG-I virus sensing	5	[37]
*k*_*IFN*,*V(TLR)*_	9.746	hr^-1^	Maximal rate of IFN production via TLR virus sensing	5	
*K*_*IFN*,*V(TLR)*_	12.511	[unitless]	Apparent dissociation constant of TLR virus sensing	5	
*k*_*IFN*,*IRF7P*_	13.562	hr^-1^	Rate of IFN production via IRF7P	5	
*Q*_*IFN*_	10.385	hr^-1^	Coefficient of export of IFN to the extracellular environment	5,10	
*k*_*STATP*,*IFNe*_	675.323	μM hr^-1^	Maximal rate of STAT phosphorylation via IFN_e_	6	
*K*_*STATP*,*IFNe*_	80.353	μM	Michaelis-Menten constant for STAT phosphorylation via IFN_e_	6	
*τ*_*STATP*_	0.3	hr^-1^	Dephosphorylation rate of STATP	6	[54]
*k*_*IRF7*,*STATP*_	0.115	hr^-1^	Rate of IRF7 induction via STATP	7	[37]
*k*_*IRF7*,*IRF7P*_	1.053	hr^-1^	Rate of IRF7 induction via IRF7P	7	
*τ*_*IRF7*_	0.75	hr^-1^	Decay rate of IRF7	7	[37,51]
*k*_*IRF7P*,*IRF7*_	0.202	hr^-1^	Rate of IRF7 phosphorylation via IRF7	8	[37]
*τ*_*IRF7P*_	0.3	hr^-1^	Dephosphorylation rate of IRF7P	8	[52]
*γ*_*H*,*V*_	0.635	hr^-1^	Rate of cell health loss due to viral load and rate of transition from virus releasing (I2) to dead (D) cells	3,9	[37]
*k*_*V*,*V*_	1.537	hr^-1^	Rate of viral replication	4	[25,37]
*K*_*V*,*IFNe*_	0.020884	μM	Michaelis-Menten constant for IFN_e_ inhibition of viral replication	4	[37]
*Q*_*V*_	0.197	PFU mL^-1^ hr^-1^	Coefficient of the rate of viral release into the extracellular environment	4,11	
*n*	3	[unitless]	Hill coefficient of TLR virus sensing	5	
*β*	1E3	mL PFU^-1^ hr^-1^	Rate of transition from uninfected (U) to eclipse phase (I1) cells	1	[38]
*τ*_*I1*_	0.167	hr^-1^	Rate of transition from eclipse phase (I1) to virus releasing (I2) cells.	2	(25)
*τ*_*Ve*_	0.542	hr^-1^	Decay rate of virus in the extracellular environment	11	
*τ*_*IFNe*_	3.481	hr^-1^	Decay rate of IFN_e_ in the extracellular environment	10	[37]
*D*_*Ve*_	54.0	μm^2^ s^-1^	Diffusion coefficient of V_e_	11	[24,25]
*D*_*IFNe*_	54.0	μm^2^ s^-1^	Diffusion coefficient of IFN_e_	10	[50,53]
*L*_*Ve*_	0.09	μm	Calculated diffusion length of extracellular virus		
*L*_*IFNe*_	0.23	μm	Calculated diffusion length of IFN_e_		
Voxel Width	3	μm	Width of lattice voxels		[29]*
Cell Size	9	μm	Width of cells		

### Diffusion of extracellular species and implementation in CC3D

Virus releasing cells (I2) release intracellular virus into the extracellular environment. Uninfected, eclipse phase and virus releasing cells (U, I1, and I2) produce and export type-1 interferons in response to virus sensing proteins and/or autocrine/paracrine signaling. In cell culture, these extracellular species diffuse freely in the medium above the apical surface of cells.

The MSIS model contains a cell lattice next to two chemical field lattices (described above) and the diffusion of extracellular species across either chemical field lattice is unaffected by the presence of cells in the adjacent cell lattice. Eq 10 models diffusion of extracellular interferons, where *D*_*IFNe*_ is the diffusion coefficient of interferon, *Q*_*IFN*_ is the rate constant for export of interferon by cells into the extracellular environment, and IFN is the internal amount of interferon inside each cell. Cell types U, I1, and I2 can produce and export interferon.

Eq 11 models diffusion of the extracellular virus, where *D*_*Ve*_ is the diffusion coefficient of virus and *Q*_*v*_ is the secretion rate constant for release of virus by late infected (I2) cells. Intracellular virus (V) is a normalized, unitless quantity representing the per cell viral load, while extracellular virus (V_e_) has units of PFU mL^-1^ and represents the concentration of infectious virus in the extracellular environment. The unit conversion is achieved via *Q*_*v*_’s units of PFU mL^-1^ hr^-1^.

Extracellular Species

$$\frac{\partial [{IFN}_{e}]}{\partial t}=D_{IFNe}\nabla^{2}[{IFN}_{e}]+Q_{IFN}[IFN]-\tau_{IFNe}[{IFN}_{e}]$$ (10)

$$\frac{\partial [V_{e}]}{\partial t}=D_{V}\nabla^{2}[V_{e}]+Q_{V}[V]-\tau_{Ve}[V_{e}]$$ (11)

CompuCell3D solvers use a simple time-slicing algorithm. Each CompuCell3D time step represents 10 minutes. CompuCell3D first calculates the integrated amount of V_e_ and IFN_e_ directly above each cell to calculate [V_e_]_*per cell*_ and [IFN_e_]_*per cell*_ and passes these values to Eq 1, 4, and 6. It then integrates the diffusion and the intracellular species’ rate equations forward in time independently, using these fixed values of [V_e_]_*per cell*_ and [IFN_e_]_*per cell*_ for the equivalent of 10 minutes. It then calculates the amount of virus released and the amount of IFN exported from each cell over 10 minutes and adds the amount released divided by the cell area into each voxel in the appropriate field at each position corresponding to a voxel of that cell. It then evaluates the probabilities for cell type transitions for each cell following Eq 1 – 3 to determine whether each cell experiences such a transition. For a more complete description of how CC3D implements simulations, please see [29].

### Initial and boundary conditions

All simulations use periodic boundary conditions along the *x* and *y* axes. To simulate high MOI conditions, all cells are initially I2 type. When simulating low MOI conditions, at time zero all cells are U type, except for one I1 cell at the center of the simulation. Table 1 gives the initial conditions for the intracellular variables of each cell type at time zero. In all simulations, the extracellular environment initially contains no V_e_ or IFN_e_.

To simulate interferon pretreatment, the simulation starts at 12 hours pre-infection, with all cells U type (initial conditions listed in Table 1) and exposed to IFN_e_ at 0.04 μM. At time = 0 hours (12 hours after IFN_e_ exposure), we simulate washing of the cells by setting IFN_e_ = 0 μM and initiate the infection by setting a cell at the center of the simulation’s lattice to the I1 type. Due to the IFN pretreatment, all cells have the same intracellular state at time zero (shown in Table 3) except for the single I1 cell, for which V is set to 6.9E-8 (unitless) [37].

**Table 3:** Intracellular chemical concentrations in cells 12 hours after *in silico* exposure to IFN_e_. These values provide the initial conditions for IFN prestimulation simulations.

Species	Initial Conditions
IFN	0.035 μM
IRF7	0.097 μM
IRF7P	0.028 μM
STATP	0.714 μM

### Parameter determination

Many MSIS model parameters come directly from the Weaver model [37]. The Weaver model was parameterized using the lowest sum-of-squares error resulting from a parallel tempering Markov chain Monte Carlo fit to data collected from HBECs infected with wild-type A/Puerto Rico/8/1934 Influenza A [49]. Each cell’s ODE model in the MSIS model is the Weaver model, modified as described previously. We adopted additional parameters from the literature [31,50–52]. Table 2 gives a comprehensive list of model parameters and their origin. Virus diffusion coefficients can vary by several orders of magnitude depending on media type, based primarily on the medium’s viscosity [24]. We set the diffusion coefficient for both V_e_ and IFN_e_ to 54.0 μm^2^ s^-1^, within the range of experimental measurements [50,53] for both species. For these diffusion coefficients, the baseline parameter set led to continuous plaque growth. We rescaled the cell type transition parameter *β* [38] from units of median tissue culture infectious dose (TCID_50_^-1^ hr^-1^) to plaque-forming units (mL PFU^-1^ hr^-1^) for consistency with the Weaver model’s units for viral load.

### Plaque growth metrics

Viral plaques are visible areas of dead or damaged cells that occur where a virus has spread across a continuous patch of cells in cell culture. At early times, a growing plaque consists of a central domain of I2 cells surrounded by a ring of I1 cells. At later times, the plaque consists of a domain of dead cells surrounded by a ring of I2 cells, in turn, surrounded by a ring of I1 cells. We measure the radial growth speed of the outer border of the domain of eclipse (I1), virus releasing (I2), and dead (D) cell types. In the simulations, we determine these speeds by seeding a single I1 cell in the center of a simulated sheet of cells and measuring the total area of each cell type over time. We assume the plaques are circular to estimate their radius. The change in the outer radius of the domain of each cell type over time gives the plaque growth velocities. Simulations involve probabilistic infection events and stochastic cell type transitions. We averaged plaque growth metrics over 20 simulations for each parameter set (S1 Fig). In experiments, plaque-plaque interference occurs when two or more plaques grow into the same spatial region, slowing the radial growth of the colliding plaques. This paper simulates only the growth of isolated plaques.

## Results

### Multicellular spatial model of RNA virus infection and IFN signaling (MSIS model) reproduces ODE model dynamics for high MOI infection

We first checked whether the MSIS model reproduced the dynamics of the Weaver model for the same simulated experimental conditions [37]. The Weaver model was fitted to data from HBECs [49] that were uniformly infected with an influenza virus at MOI = 5. For such high MOI initial conditions, the spatial inhomogeneity of the multiscale model should have a negligible effect on the population-level dynamics, because all cells are infected simultaneously.

**Fig 2:**
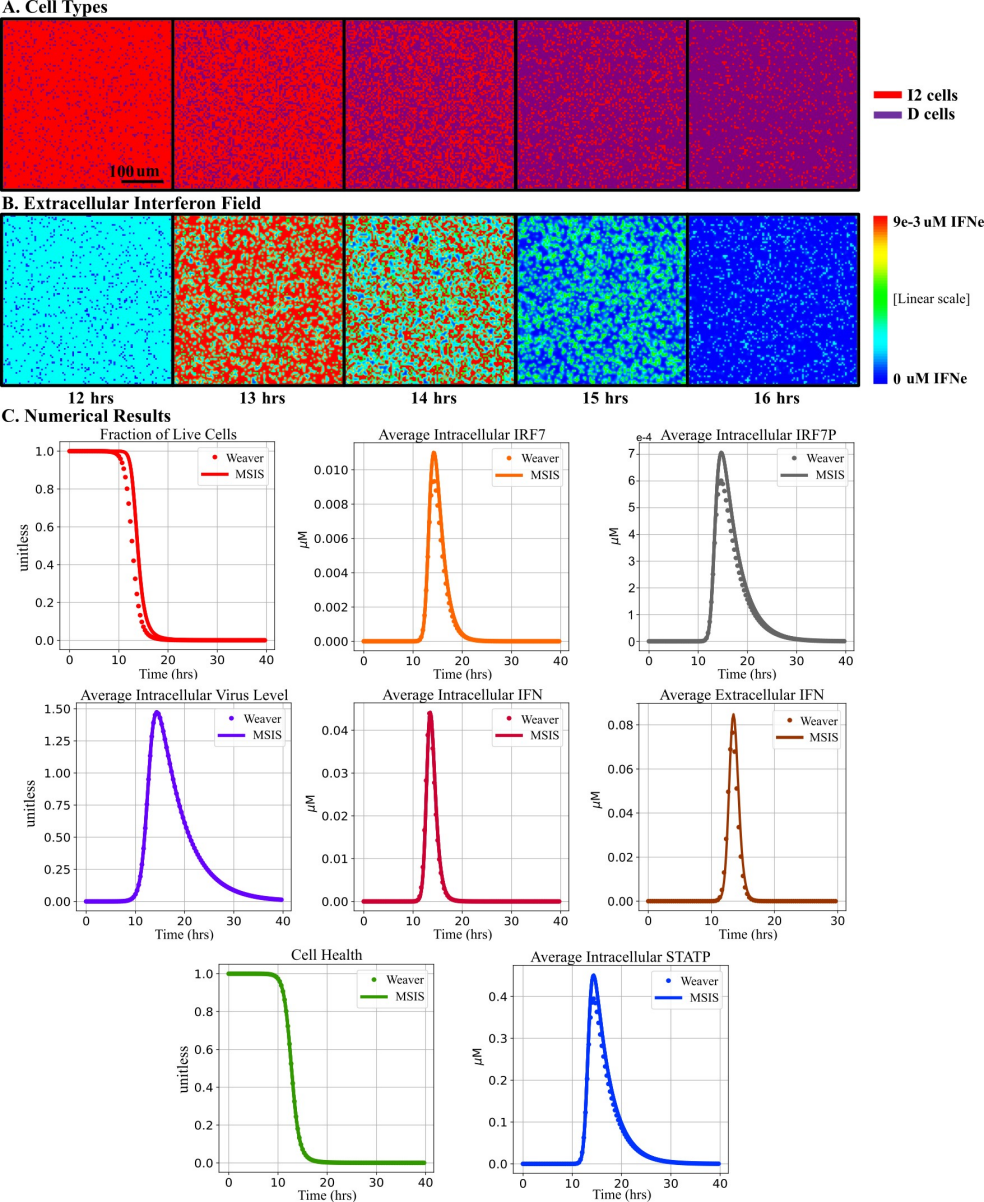
Comparison of time series for key variables between the multicellular spatial interferon signaling (MSIS) model and Weaver model for high MOI. All cells are initially infected with 6.9E-8 (unitless) virus, matching the original data to which the Weaver model was fit [49]. (A) Snapshots of the cell field showing cell type (virus releasing [I2] in red and dead [D] in purple) at different times in a representative MSIS simulation. (B) Snapshots of the concentrations of extracellular interferon (high concentrations in red, low concentrations in blue) at different times in a representative MSIS simulation. (C) Time series for key variables for the Weaver and MSIS models. MSIS simulations are averaged over 20 replicas at matching times (S1 Fig). Error bars are included but are too small to be visible. For the MSIS model, average concentrations for intracellular species and Health are calculated over all live (I2) cells at each time point while the Average Extracellular IFN for the MSIS model is the average of IFN_e_ across the entire simulation domain.

The average concentrations of the intracellular species and viral titers of the MSIS model are similar to those of the Weaver model under high MOI conditions. For MOI = 5, more than 99% of cells are expected to be infected. The Weaver model has two cell types, alive and dead, and does not include eclipse phase cells. To replicate the Weaver model simulations for an MOI = 5 infection, we initialized the MSIS model with only virus releasing (I2) cells and no eclipse phase (I1) cells [24]. A non-uniform cell type distribution (Fig 2A) and local IFN_e_ concentration field (Fig 2B) emerge in the MSIS model simulations due to the stochastic cell transitions, which lead to spatially varying IFN_e_ and V_e_, which in turn lead to non-uniform rates of death of I2 cells.

Fig 2C compares the average fraction of live cells and average levels of chemical species in the MSIS and Weaver models. The fraction of live cells vs time has the same shape in the two models, but dead cells start accumulating slightly later in the MSIS model than in the Weaver model. A major distinction between the MSIS model and the Weaver model is that MSIS cells are discrete. Dead cells have no intracellular chemical species and do not release virus or export IFN. These distinctions mean that we must compare the levels of intracellular chemical species (or Health) in live cells (I2 cells in this case) to the Weaver model outputs. However, the levels of extracellular species (IFN_e_) reflect production by all cells over time and thus we compare the IFN_e_ averaged over all lattice sites to the Weaver model outputs. For homogeneous, high MOI starting conditions, all concentrations grow rapidly after the onset of viral release, reach a maximum, and then decay nearly exponentially on a slower time scale. For each variable, the MSIS model value is always greater than or equal to the Weaver model value. Relative errors are largest at times when the values are near their maxima and are always less than 15%. Cell death begins slightly later in the MSIS model than in the Weaver model and the cell death rate increases slightly faster, so that all cells die at nearly the same time. Since the MSIS model produces dynamic responses like those of the Weaver model under high MOI, we will assume that differences between the dynamics of the two models at low MOI result from spatial effects, not from differences in parameters or errors in spatializing the Weaver model. This paper focuses on simulated spatially heterogeneous low MOI initial conditions, which more closely resemble *in vitro* plaque growth assays than high MOI.

### MSIS model recapitulates experimentally observed plaque formation and growth dynamics

**Fig 3:**
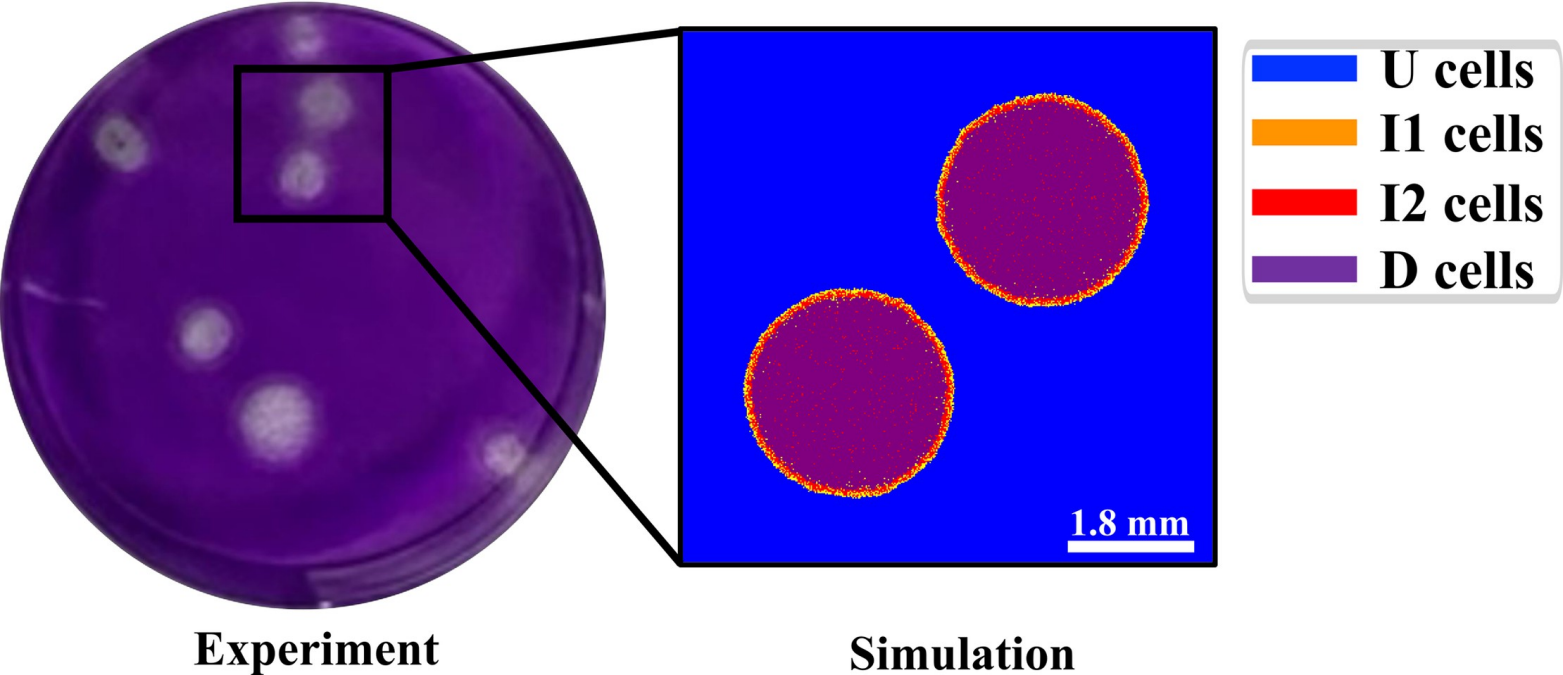
Comparison of an experimental plaque assay for influenza (H5N1; left) with an MSIS model plaque simulation (for H1N1; right). The simulation seeded two plaques in a simulation domain to replicate a subset of the experimental area. The simulated plaques have a similar structure to the experiment. Outlined area in the experimental image corresponds roughly to the area of the simulation domain.

High MOI experiments are useful for determining the time course of viral titer and how long cells survive a viral infection, but, unlike plaque assays, they do not provide information about viral spread and the spatial aspects of cytokine responses. We explored low MOI plaque assay experiments *in silico*. Fig 3 (left) shows multiple plaques that formed in a culture of cells infected with an H5N1 influenza virus. We first evaluated if the MSIS model produced plaque-like structures beginning with a single point of infection, similar to those in experiments for low MOI. We created a simulation with two I1 cells seeded in similar locations to a subset of the plaques shown in Fig 3‘s left image. Fig 3 right shows the simulation at 80 hours. The MSIS model reproduces the circular geometry of experimental plaques. The length scales differ between the experimental and simulated plaques because the MSIS model is parameterized for an H1N1 virus, while the experiment shown used a faster replicating H5N1 virus.

**Fig 4:**
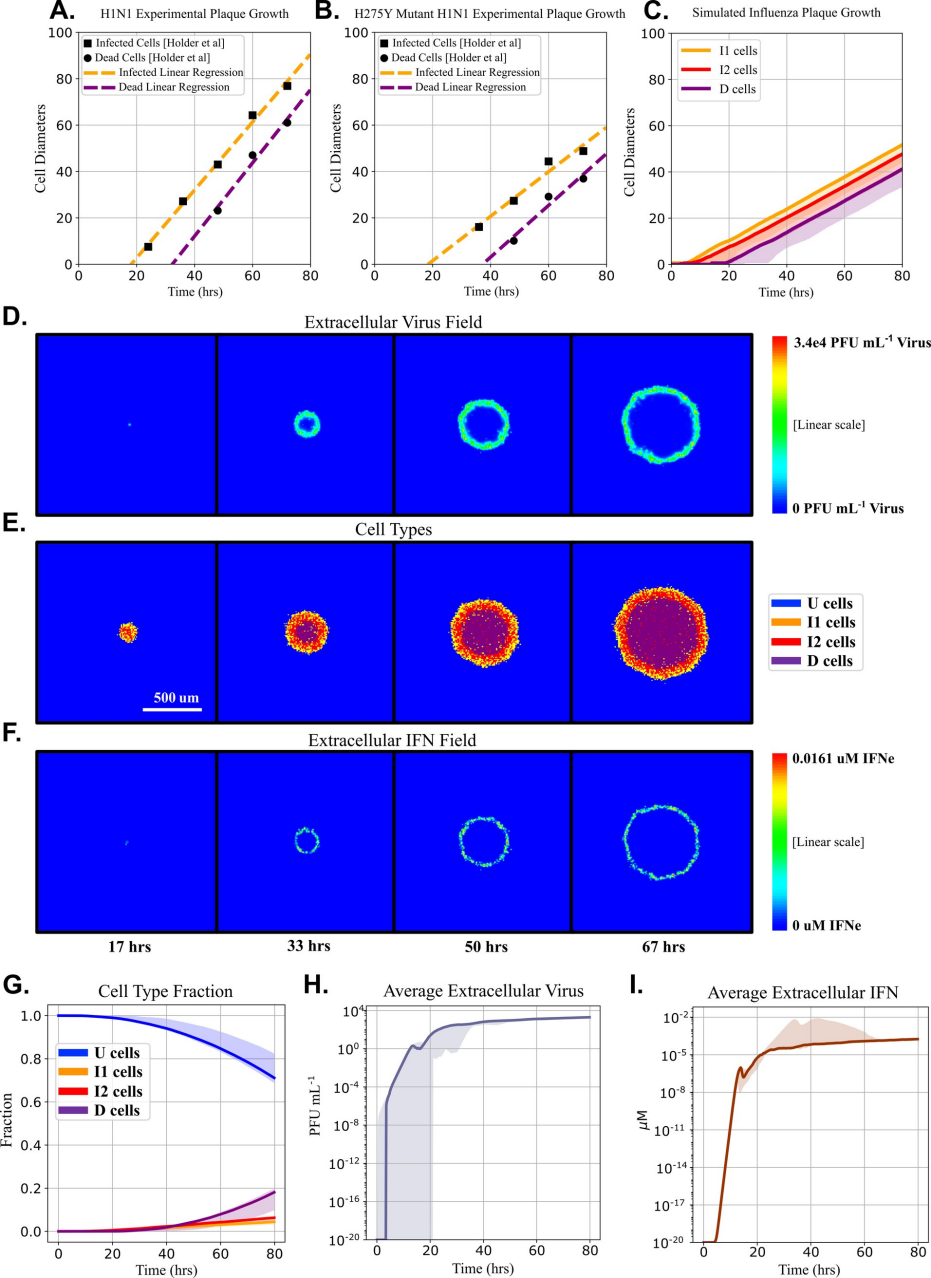
Plaque growth simulations replicate experimentally observed linear radial plaque growth. (A, B) Radius vs time of outer boundaries of the domains of infected and dead cells for wild-type and H275Y mutant A/Miss/3/2001 (HIN1) infection-induced plaques, respectively. Data reconstructed from [24]. Squares indicate the radius of the outer edge of the plaque (the boundary between infected cells and uninfected cells) and circles indicate the radius of the boundary between dead cells and infected cells in the plaque. Dotted lines show a linear regression for visualization of plaque radius vs time. (C) Simulated plaque growth shows the lag phase and linear growth of the experimental plaques. The solid line indicates the median for 20 simulation replicas (S1 Fig includes additional information on the simulations standard deviations) and the shaded areas indicate the 5^th^ and 95^th^ percentiles of observed values. Fig 4D, 4E, and 4F show sequential snapshots (at 17, 33, 50 and 67 hours) of the V_e_ field (D), cell type (E), and IFN_e_ field for a single simulation replica of a growing plaque. Time progresses from left to right. Fig 4G, 4H, and 4I show the median (solid line) and 5^th^ to 95^th^ percentile (shaded areas) of the simulated cell types, average V_e_, and IFN_e_, respectively, calculated for an ensemble of 20 simulation replicas (S1 Fig).

Next, we explored plaque growth dynamics in the MSIS model. Fig 4A and 4B show experimental plaque radii vs time (data reproduced from [24]). While the increase in viral load during infection is typically exponential, plaque radius grows linearly in time. The experiment measured the radius of the outer edge of the domain of dead cells (equivalent to D in the model) and the outer edge of the domain of infected cells (equivalent to I1 in the model). The MSIS model distinguishes the eclipse phase (I1) from virus releasing (I2) cells, which normally cannot be distinguished in experimental plaque growth assays. For simulations beginning with a single I1 cell, Fig 4C shows that the MSIS model replicates several experimental observations. Both experiments (Fig 4A and 4B) and the MSIS model (Fig 4C) show a lag phase with no plaque growth. During the lag phase, the cells are not releasing virus and no new cells are being infected. The simulation follows Tables 1 and 2 for initial conditions and model parameters, respectively. Fig 4E shows snapshots of the cell types at 17 hours, 33 hours, 50 hours, and 67 hours in a single replica simulation. The plaque consists of a central nearly round disk of dead cells, surrounded by a concentric ring of I2 cells, in turn, surrounded by a concentric ring of I1 cells. A few I1 and I2 cells are scattered in the dead cell disk, dead and I1 cells in the I2 ring, and I2 cells in the I1 ring. Fig 4D shows snapshots of the V_e_ field at corresponding times in the same simulation replica. The virus concentration is maximal over the ring of I2 cells and decreases rapidly at larger and smaller radii. Fig 4F shows the IFN_e_ concentration field at corresponding times for the same simulation replica. Fig 4F shows that the IFN_e_ level is high in a very narrow ring over the boundary between the I1 and I2 rings in the plaque. Fig 4G shows the cell type composition of the culture over time. Dead cells first appear after 20 hours, after which the radius of plaque’s central, circular domain of dead cells increases linearly in time. The radial growth rate of the plaque remains constant until the plaque reaches the edge of the simulation domain. Around 18 hours post-infection, V_e_ (Fig 4H) and IFN_e_ in the culture (Fig 4I) decrease briefly because the initially infected cell has died and stopped releasing virus and exporting IFN. During this time, the second generation of infected cells (those infected by the virus released by the initially infected cell) are primarily I1 phase and not yet releasing virus. The MSIS model recapitulates the experiments’ linear radial growth of viral plaques. The MSIS model’s ability to simulate both high and low MOI experiments and reproduce phenomena seen experimentally, without additional parameter fitting to these conditions, gives confidence in its predictive capabilities in the novel circumstance of low MOI simulations. The next five sections of Results are based on simulations of plaque growth assays, which give insights into the significance of spatial inhomogeneity to the mechanisms regulating plaque growth.

### Increased STAT activity leads to arrested plaque growth and reduces final plaque diameter

**Fig 5:**
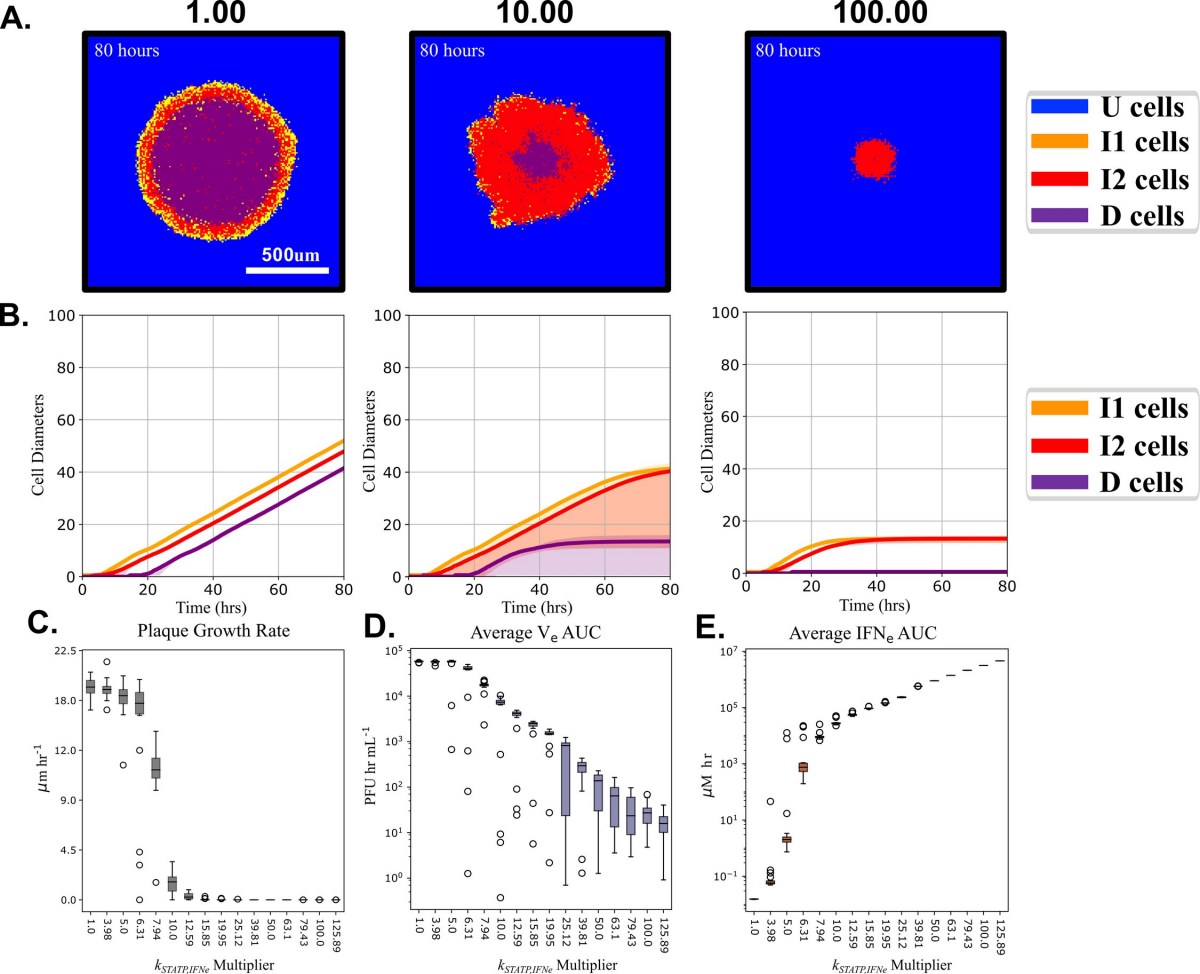
Elevated STATP activity (larger *k*_*STATP*,*IFNe*_) leads to arrested plaque growth. (A) Images of the simulated plaques at 80 hours post-infection for a single simulation replica when *k*_*STATP*,*IFNe*_ was 1x, 10x, or 100x larger than its baseline value. Arrested plaque growth occurs when *k*_*STATP*,*IFNe*_ is 10x or 100x larger than baseline. (B) The median (solid line) and 5^th^ and 95^th^ percentiles (shaded regions) for 20 simulation replicas of the cell types over time for *k*_*STATP*,*IFNe*_ at 1x, 10x, or 100x larger than its baseline value of 45.9 μM hr^-1^. (C) The plaque radius’ linear growth rate at 80 hours, (D) the area under the curve (AUC) of the average V_e_, and (C) the AUC of the average IFN_e_ when *k*_*STATP*,*IFNe*_ is changed between its nominal value to 125.98x nominal, over 20 simulation replicas.

The JAK/STAT pathway triggers an inflammatory reaction via auto/paracrine signaling and inhibition of this pathway has been implicated in improved H1N1 influenza survival in mice [55]. We wished to assess the impact of STATP activity on plaque growth dynamics in the MSIS model. We simulated plaque growth while altering the ability of extracellular interferons to activate the JAK/STAT pathway in the MSIS model by increasing the value of *k*_*STATP*,*IFNe*_ (Eq 6 and Table 2) from its baseline value (45.9 μM hr^-1^, Table 2) up to 125.89x this value. For three values of *k*_*STATP*,*IFNe*_, we show the plaque size and shape at 80 hours post-infection (Fig 5A) and the cell type dynamics over time (Fig 5B). The baseline value leads to unconstrained plaque growth. Values of *k*_*STATP*,*IFNe*_ *≥* 459.22 μM hr^-1^ (10x baseline value) led to the arrest of plaque growth. Increasing *k*_*STATP*,*IFNe*_ *≥* 4592.2 μM hr^-1^ (100x baseline value) reduces the time to plaque growth arrest, resulting in smaller plaques. These simulations use Table 1 initial conditions and Table 2 parameters except for the modified values of *k*_*STATP*,*IFNe*_. Increasing the degree to which IFN_e_ promotes STATP production arrests plaque growth and reduces the final plaque size.

Fig 5C shows the rate of change of plaque radius at the end of the simulation as a function of *k*_*STATP*,*IFNe*_, which controls the degree to which a given level of IFN_e_ leads to active STATP, Eq 6. For *k*_*STATP*,*IFNe*_ multipliers of 15.85 and above, the plaque growth rate is always zero at the end of the simulation, indicating plaque arrest. Arrest occurs earlier for higher *k*_*STATP*,*IFNe*_ (Fig 5B, 2nd and 3rd panels). *k*_*STATP*,*IFNe*_ multipliers above 6.31 reduce the area under the curve (AUC) for average V_e_ (Fig 5D), while multipliers between 1.0 and 6.31 have little to no effect on viral AUC. The AUC of average IFN_e_ (Fig 5E) increases with increasing *k*_*STATP*,*IFNe*_, with a dramatic increase in the range of multipliers of 6.31 to 10.0. Note logarithmic ordinate scale for both average V_e_ and IFN_e_ AUC. Larger *k*_*STATP*,*IFNe*_ would correspond to a stronger interferon response and reduced viral titer. *k*_*STATP*,*IFNe*_ *≥* 4592.2 μM hr^-1^ leads to non-physiological unbounded production of IFN, due to the lack of an IFN-mediated cell death mechanism in both the Weaver and MSIS models.

### Elevated RIG-I activity delays cell death and increases IFN production

**Fig 6:**
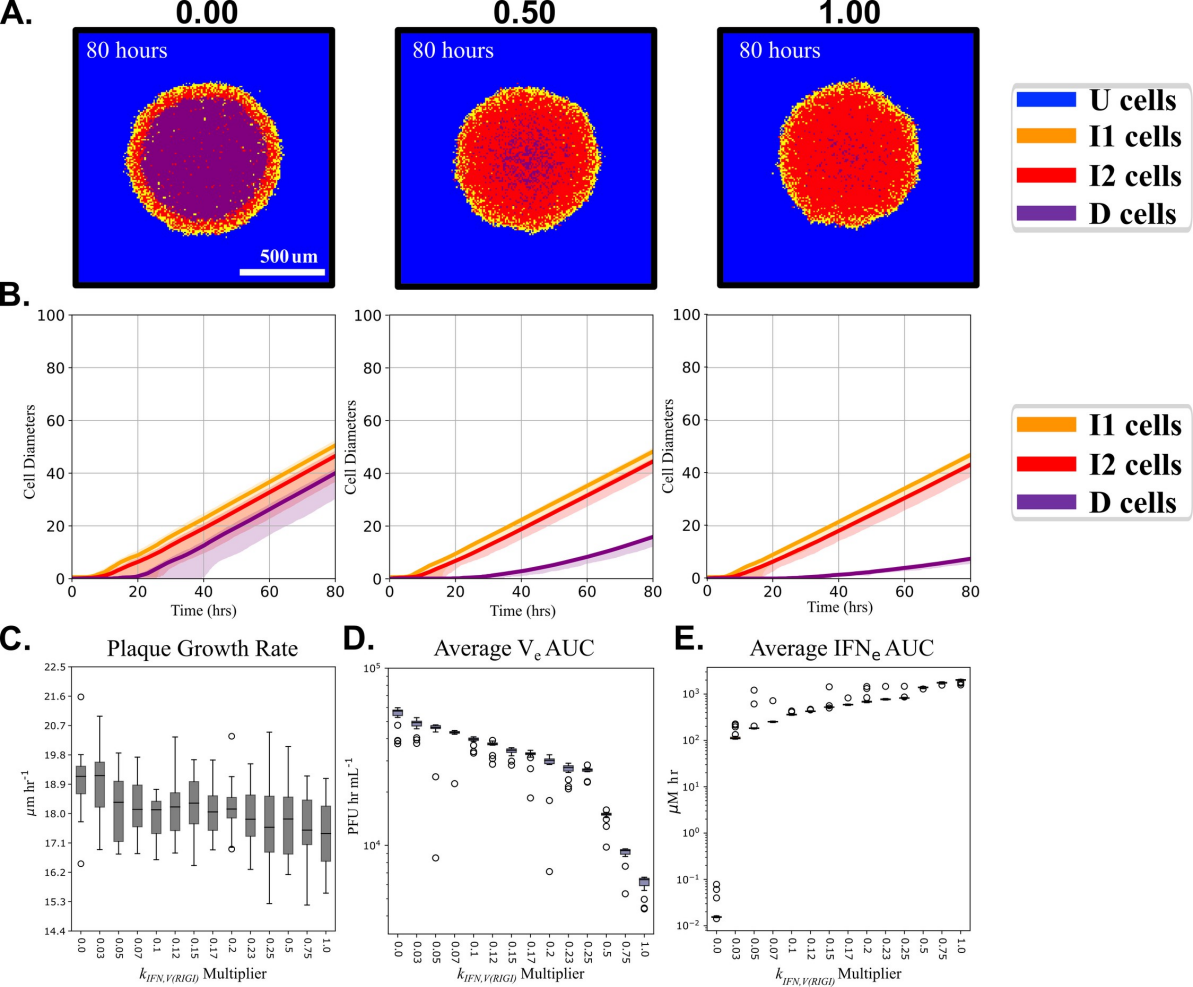
Increased RIG-I activity (*k*_*IFN*,*V(RIGI)*_) lowers plaque growth rates and viral titers, slows cell death, and increases interferon production. (A) Images of plaques at 80 hours post-infection for a representative simulation replica for three values of *k*_*IFN*,*V(RIGI)*_ (0.5E5 μM hr^-1^and 10E5 μM hr^-1^) and (B) the median (solid line) and 5^th^ and 95^th^ percentiles (shaded regions) of the plaque radius over time for 20 simulation replicas for *k*_*IFN*,*V(RIGI)*_ equal to 0x, 0.5x, or 1x its nominal value of 10E5 μM hr^-1^. (C) The plaque growth rate at 80 hours, (D) the area under the curve (AUC) of the average V_e_, and (E) the AUC of the average IFN_e_ for different values of *k*_*IFN*,*V(RIGI)*_, over 20 simulation replicas. Full data with 5 additional outliers for the plaque growth rate and the average V_e_ AUC are available in S2 and S3 Figs, respectively.

In influenza infection, greater viral inhibition of RIG-I signaling via NS1 protein often increases viral infection severity [56–58]. We wished to investigate the effects of decreasing this antagonistic strength on plaque growth dynamics *in silico*. In our simulations *k*_*IFN*,*V(RIGI)*_ controls the strength of the RIG-I response, with larger values corresponding to a stronger response (more IFN produced per unit of virus, Eq 5). Our simulations so far assumed that the invading virus completely inhibited the RIG-I pathway (*k*_*IFN*,*V(RIGI)*_ = 0, Eq 5). Previous work used data from cells infected with an NS1-knockout influenza virus (A/Puerto Rico/8/1934 [dNS1PR8]) to estimate the rate of IFN production via RIG-I virus sensing (*k*_*IFN*,*V(RIGI)*_ = 10E5 μM hr^-1^ [37]). We ran single-plaque growth simulations for 14 values of *k*_*IFN*,*V(RIGI)*_ between 0% and 100% of this estimate. These simulations use Table 1 initial conditions and Table 2 parameters except for the value of *k*_*IFN*,*V(RIGI)*_. At 80 hours post-infection (Fig 6A) the plaque radius is nearly the same for all cases, shown for 0%, 50%, and 100% activity. However, the cell type composition of the plaque (Fig 6B) differs significantly, with significantly less cell death and thus a higher fraction of I2 cells, for *k*_*IFN*,*V(RIGI)*_ multipliers greater than 50%. Higher levels of RIG-I signaling (larger values of *k*_*IFN*,*V(RIGI)*_) only slightly reduce the radial plaque growth at the end of the simulations (Fig 6C). The AUC of the average V_e_ decreases steadily with increasing RIG-I activity (Fig 6D), decreasing more rapidly for *k*_*IFN*,*V(RIGI)*_ multipliers greater than 25%. The AUC of average IFN_e_ increases dramatically for parameter multipliers less than 0.03x nominal and more gradually thereafter (Fig 6E).

Increasing levels of RIG-I activity for a given level of virus (larger *k*_*IFN*,*V(RIGI)*_) increases the intracellular production of IFN (Eq 5). This higher intracellular IFN leads to higher IFN_e_ due to cell export (Eq 11). Higher IFN_e_ leads to a reduction of intracellular viral levels (Eq 4). Since the rate of decrease of cell health (H) is linear with respect to intracellular virus level (Eq 9), and the death rate of cells is proportional to both the virus level and H (Eq 3), higher values of *k*_*IFN*,*V(RIGI)*_ increase the survival time of infected cells both by decreasing the intracellular virus level and by slowing the decrease of H. Overall, the model predictions are consistent with the expectations that greater RIG-I activity leads to reduced virus production (i.e. reduced virus titers).

### Interferon prestimulation arrests plaque growth

In experiments, prestimulation of cell cultures with type-I interferons reduces the amount of virus produced in cells infected with SARS-CoV, SARS-CoV-2 [16], or influenza [17]. We simulated prestimulation experimental conditions in the MSIS model to explore these protective effects by exposing uninfected (U) cells to IFN_e_ at 0.04 μM at 12 hours pre-infection (-12 hours, since infection is referenced as time = 0), using the values of the parameters in Table 2. All cells were exposed to the same concentration of IFN_e_. Since cell type transitions do not occur in the absence of virus, after 12 hours, all cells had identical intracellular chemical concentrations shown in Table 3. At 0 hours, IFN_e_ is set to zero to simulate washing IFN_e_ out of the cell culture, and a single cell is infected *in silico* by setting it to the I1 type. We then assessed the impact of IFN prestimulation on plaque growth.

**Fig 7:**
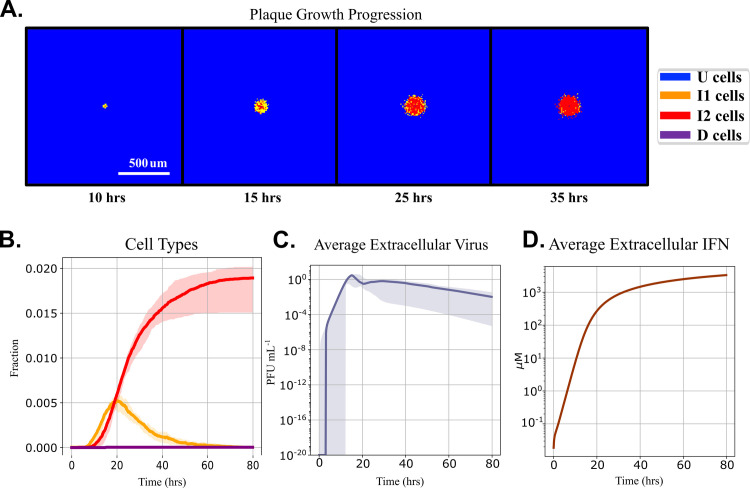
Prestimulating cells with type-I interferon led to plaque growth arrest in simulations. We simulate an experiment with 0.04 μM IFN_e_ prestimulation for 12 hours, which is removed immediately before infection. (A) Sequential snapshots (at 10, 15, 25 and 35 hours post-infection) of plaques for a representative simulation replica. (B) Cell type fractions vs time. (C) Average V_e_ vs time and (D) the average IFN_e_ vs time. The solid lines indicate medians and shaded areas represent the 5^th^ and 95^th^ percentiles over 20 replicas.

Simulated prestimulation entirely arrests plaque growth after 35 hours (Fig 7A), while the same initial infection in a field of naïve, unstimulated cells resulted in the infection and eventual death of all simulated cells (Fig 4C and 4G). Only the initially infected cell dies. The proportion of eclipse phase (I1) cells steadily decreases after 20 hours, indicating a cessation of new infections (Fig 7B). The average V_e_ concentration (Fig 7C) also decreases after 20 hours. The average IFN_e_ concentration (Fig 7D) is higher than in the baseline simulation. Time series for the intracellular variables, akin to Fig 2C, are available in S4 Fig.

### Faster interferon diffusion promotes plaque growth arrest

**Fig 8:**
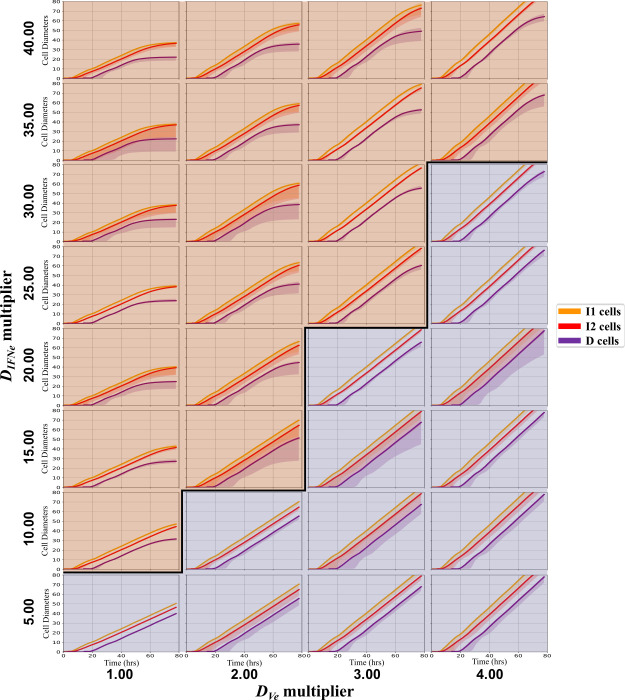
Dependence of plaque growth rate and arrest on viral and IFN diffusion coefficients. Each box shows 20 replica simulations’ cell type progression over time for the indicated diffusion coefficient multiplier combination. The solid-colored lines indicate the medians of the radii, and the shaded regions indicate the 5^th^ and 95^th^ percentile radii over 20 replicas. In the orange shaded region (above the bold line) plaques arrest by 80 hours. In the blue shaded region (below the bold line), plaques continue to grow until the end of the simulation.

Diffusion coefficients for the virus and interferon will depend on virion diameter and the viscosity and chemistry of the medium *in vitro* [24]. We varied virus and interferon diffusion coefficients simultaneously. Because the actual diffusion coefficient of the extracellular IFN is likely to be 11x to 17x greater than that of the virus, we varied the interferon diffusion coefficient from 54 μm^2^ s^-1^ to 2160 μm^2^ s^-1^ (1x to 40x the baseline interferon diffusion coefficient) and the virus diffusion coefficient from 54 μm^2^ s^-1^ to 216 μm^2^ s^-1^ (1x to 4x the baseline virus diffusion coefficient). Simulations used initial conditions from Table 1 and parameters from Table 2 except for the revised diffusion coefficients. We calculated the median growth rate of the plaque radius at the end of the simulation over 20 replicas. If the median linear growth rate was 0 at the end of the simulation, we classified the parameters as leading to plaque arrest (orange); otherwise, we classified the parameters as leading to continued growth (blue). In our simulations, an interferon diffusion coefficient of 8x to 10x the viral diffusion coefficient led to plaque growth arrest (Fig 8). The curved boundary between the domains suggests that for high viral diffusion coefficients, virus diffusion ceases to be the rate-limiting factor in plaque growth. In summary, there is a broad range of values for both diffusion coefficients in which plaque arrest and continuous growth may occur. Better estimates of these diffusion coefficients can help clarify the relative importance of intracellular versus extracellular processes in viral infection.

### Sensitivity analysis reveals that the main parameters controlling radial plaque growth differ between regimes

**Fig 9:**
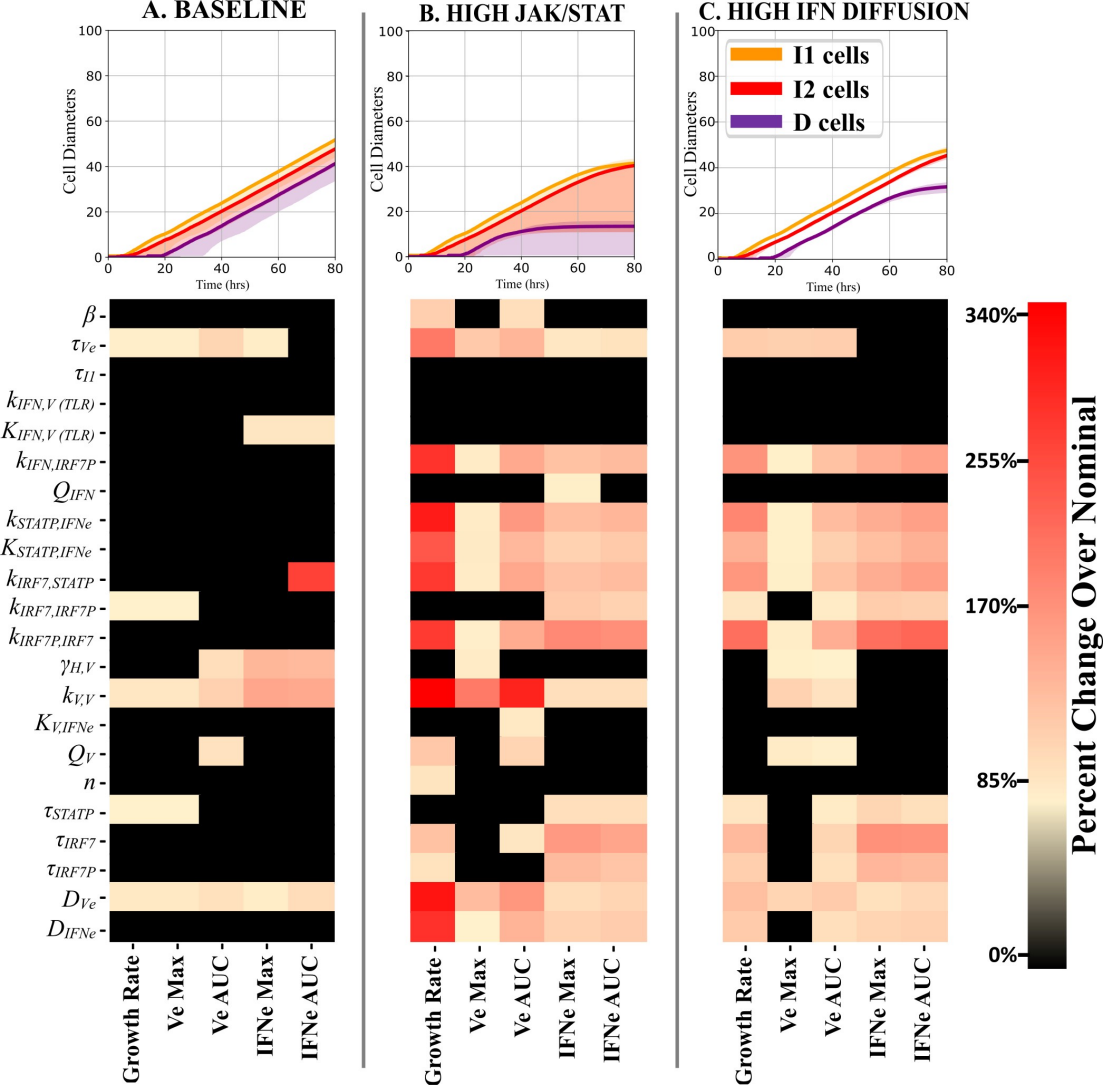
Local single-factor sensitivity analysis for three simulation regimes. (A) “Baseline” corresponds to the baseline parameters in Table 2. (B) “High JAK/STAT” corresponds to a 15x increase in the phosphorylation rate of STATP via the JAK/STAT pathway (parameter *k*_*STATP*,*IFNe*_), with all other parameters as in Table 2. (C) “High IFN Diffusion” corresponds to a 10x increase in the diffusion coefficient of IFN_e_, with all other parameters as in Table 2. Sensitivity analyses varied each parameter one-at-a-time ± 25% around its unperturbed value and quantified the average plaque radius growth rate at the end of the simulation, the maximum extracellular virus (V_e_) and interferon (IFN_e_) levels that occurred, and the area under the curve (AUC) for both average V_e_ and IFN_e_. The sensitivity metrics average the absolute values of the metric for increased and decreased parameters over 20 replicas for each parameter set.

To determine how individual parameters affect the growth of plaques, we performed local sensitivity analyses around parameter sets in three regimes in parameter space; the baseline parameter set (Table 2), the High JAK/STAT regime (*k*_*STATP*,*IFNe*_ = 688.5 μM hr^-1^, 15x baseline value, all other parameter values as in Table 2), and the High IFN Diffusion regime (*D*_*IFNe*_ = 540.0 μm^2^ s^-1^, 10x baseline value, all other parameter values as in Table 2). For each regime, we ran 20 simulation replicas using the regime’s nominal parameter values. Then, we perturbed each parameter individually ±25%, ran 20 simulation replicas for each perturbed parameter set, and performed statistical analyses on several sensitivity metrics derived from the simulated trajectories. Sensitivity metrics include the percent change from the average of the baseline simulations of the plaque radius growth rate, the maximum value of V_e_ and IFN_e_ that occurred over time, and the AUC of average V_e_ and IFN_e_. We determined the statistical significance of the change in each metric from its unperturbed value using a Student’s t-test. Statistical test results and sensitivity metrics are reported in the Supporting Information (S5, S6, and S7 Figs). Increasing and decreasing the parameter values primarily led to directionally consistent changes in the sensitivity metrics (e.g., if the metric increased when the parameter increased by 25%, then the metric also decreased when the parameter decreased by 25%). The top row of Fig 9 shows the cell type progression for plaque growth assays for each regime.

Previous sections demonstrated that variation in multiple parameters could lead to either continuous or arrested plaque growth. The baseline parameter set leads to the continuous growth of the plaque. In this regime, the rate of STATP dephosphorylation (determined by *τ*_*STATP*_, Eq 6), the strength of induction of IRF7 by STATP and IRF7P (determined by the *k*_*IRF7*,*STATP*,_ and *k*_*IRF7*,*IRF7P*_ rate parameters in Eq 7), the maximal rate of viral replication (represented by the rate parameter *k*_*V*,*V*_ in Eq 4), the extracellular virus diffusion coefficient (*D*_*Ve*_ in Eq 10), and the rate of nonspecific extracellular viral clearance (*τ*_*Ve*_ in Eq 10) have the largest effects on the metrics. For example, increasing the maximal viral replication rate (*k*_*V*,*V*_, effect shown in S8 Fig), the probability of transition from U to I1 cell types (*β*, effect shown in S9 Fig), or the extracellular virus diffusion coefficient (*D*_*Ve*_, effect shown in Fig 8) leads to faster plaque growth, whereas increasing the virus release rate to the extracellular environment (*Q*_*V*_, results not shown) or the extracellular IFN diffusion coefficient (*D*_*IFNe*_, effect shown in S10 Fig) slows plaque growth. The High JAK/STAT and High IFN Diffusion regimes both have arrested plaque growth. In these regimes, the parameters associated with the activation of paracrine signaling have statistically significant sensitivity to perturbations tested. The magnitude of the effects is higher in the High JAK/STAT regime than in the High IFN Diffusion regime. This, combined with the difficulty in selective diffusion length modification, suggests that paracrine signaling is a more feasible target for immunomodulation. The increase in parameter sensitivity in arrested plaque growth regimes also suggests that experimental conditions leading to arrested growth could improve the parameterization of future models and investigations into the interferon signaling response to viral infection.

## Discussion

In this work, we developed a mechanistic, multicellular spatial model of interferon signaling (the MSIS model) that we used to evaluate how changes in select reaction rates impacted plaque growth in RNA virus-infected cell cultures. The MSIS model produced plaque-like structures (Fig 3). The MSIS model includes parameters that were fit to data from H1N1-infected cell culture experiments, but several parameter estimates also come from the literature (see Table 2). Without additional parameter training, we showed that the model produced plaque growth dynamics (Fig 4C) similar to those observed in cells infected with two different H1N1 influenza viruses (Fig 4A and 4B). We then focused on using the MSIS model to evaluate how altering intracellular signaling rates and/or diffusion rates might impact plaque growth and performed sensitivity analyses to determine the experimental conditions under which the model’s parameter values can best be estimated.

One of the most significant outcomes of this study is that the sensitivity analysis of the MSIS model suggests that experiments should be performed in conditions that lead to plaque growth arrest rather than unlimited growth to improve the identifiability of interferon signaling parameters (Fig 9). Often, cell culture experiments of virus growth dynamics employ cell lines or conditions that promote virus plaque growth. For example, Vero cells are frequently used in studies because they do not produce interferon and therefore support robust virus replication. However, our sensitivity analysis shows that performing experiments in cells with more robust IFN responses will provide more informative data to estimate 19 of the interferon signaling parameters, compared to only 9 parameters being significantly sensitive in regimes leading to unconstrained plaque growth. And 8 of these 9 parameters significantly affect the model outputs under both constrained and unconstrained plaque growth. In all, the model suggests that experiments performed in IFN-competent cells under conditions that lead to plaque arrest are best for accurately inferring interferon signaling-associated parameter values.

We also used the MSIS model to evaluate the effects of increased paracrine activity (via STATP), increased intracellular virus detection (via RIG-I), and prestimulating cells with IFN_e_. All three changes lead to increased concentrations of extracellular IFN but only elevated paracrine signaling resulting from enhancing STATP activation (Fig 5) and interferon prestimulation (Fig 7) led to plaque growth arrest. Both IFN prestimulation and enhanced STATP production via extracellular IFN resulted in a reduced concentration of extracellular virus and an increased concentration of extracellular IFN. The final plaque size at the end of the simulations is similar when comparing the IFN prestimulation simulations (Fig 7A) to the simulation when STATP activation via extracellular IFN is enhanced by 100x (Fig 5A; furthest panel on the right). Enhanced STATP activation and IFN prestimulation leading to suppressed virus production and plaque growth are consistent with known biology and experimental observations [59,60]. However, the model’s predictions on the effects of enhancing intracellular detection of virus via the RIG-I pathway differs significantly from experimental observations. Experiments show that enhanced RIG-I binding of viral RNA leads to increased IFN production, reduced virus production, and smaller plaques [61]. Increasing RIG-I activity *in silico* increased IFN production and decreased virus production but did not significantly change the plaque size at the end of the simulation (Fig 6A). It did affect the cell type demographics, leading to significantly fewer dead cells and many persistent I2 cells (Fig 6B). Future work could investigate the effect of intracellular IFN and viral load on the rate of cell death, which is independent of these factors in the MSIS model (Eq 3).

We then considered how diffusion coefficients impact plaque growth (Fig 8). A Stokes-Einstein estimate of diffusion coefficients for virus particles (with an effective radius of 80–120 nm) [62] and interferon proteins (with an effective radius of 7 nm) [63] predict 11x – 17x larger diffusion coefficients for interferon in most media. While diffusion coefficients can vary over several orders of magnitude during the course of a single cell culture experiment (due to cell secretion of molecular species like collagen which increase medium viscosity or proteases which can decrease it) [24], we used a constant, equal, diffusion coefficient for both species (54.0 μm^2^ s^-1^) in our baseline simulations, resulting in continuous radial plaque growth. The decay rates (*τ*_*Ve*_ and *τ*_*IFNe*_) yield effective diffusion lengths for V_e_ and IFN_e_ of 0.09 μm and 0.23 μm, respectively. In Fig 8, we explored how changing the diffusion coefficients impacts plaque growth, identifying a clear boundary between regimes of arrested and continuous plaque growth. Fig 8 shows that even when *D*_*IFNe*_ is significantly larger than *D*_*Ve*_ both arrested and unconstrained plaque growth can occur for different values of *D*_*IFNe*_. In summary, we show that the model can produce unconstrained and constrained plaque growth over a wide range of diffusion coefficient combinations. Future work will focus on refining these values and may consider time-dependent diffusion coefficients.

One major shortcoming of the MSIS model is the lack of additional mechanisms to support simulating cell death. During infection, cell death occurs via several mechanisms, including via programmed cell death (apoptosis) and pyroptosis, cell death induced via inflammasomes [64]. Lacking these mechanisms, cell death only occurs in the MSIS models as the intracellular concentration of virus increases and the cell health declines (Eq 3). But as seen in Figs 5A, 6A, and 7A, cells can become stuck in the I2 cell type as the reduced concentration of intracellular virus and the slow rate of health decline significantly reduces the likelihood of a cell transitioning to the dead type. The equation that defines how cell health declines (Eq 9) was directly translated from a population-level model where health translated to the fraction of uninfected cells. In that context, Eq 9 is reasonable, but as a model of the health of a single cell, having the rate of health decline be linearly dependent on the current health of the cell (i.e., health declines more rapidly for healthier cells) might not be reasonable. To improve the model’s relatability to experimentation, future work will focus on including additional mechanisms of cell death as well as improving the kinetic description of how cell health impacts a cell’s transition to death.

The SARS-CoV-2 and influenza viruses for which this model was constructed have many similarities. Like influenza’s NS1 protein, SARS-CoV’s NSp1 antagonizes RIG-I signaling [58], and genome analysis shows an 87% conservation of NSp1’s genome between SARS-CoV and SARS-CoV-2 [16]. This similarity suggests that the MSIS model could readily be adapted to model SARS-CoV-2-induced interferon signaling from measurements of SARS-CoV-2-specific virus kinetics. The MSIS model can also be extended to consider additional spatial aspects of infection. The modular architecture supports independent and collaborative development of extensions to account for additional immune response mechanisms in vitro such as IFN-mediated cell death. It also supports extending the model to include aspects of the immune response in vivo such as propagation of IFN signaling by local innate immune cells and recruitment of adaptive immune cells to the site of infection. The model is available for download from GitHub (https://github.com/ImmuSystems-Lab/Multicellular_Spatial_Model_of_RNA_Virus_Replication) to support further community adaptation.

## Supporting information

S1 Fig Standard deviation and standard error of simulations versus replicas for the baseline (Table 2). Used to justify n = 20 replicas for sensitivity analyses and parameter sweeps. Standard deviation (SD) is blue while standard error (SE) is orange.
(TIF)

S2 Fig Fig 6C with all outliers visible. Five outlier simulations of increased *k*_*IFN*,*V(RIGI)*_ activity over baseline resulted in fully arrested plaques by 80 hours post-infection. These outliers were cropped out in the original figure to show the distribution of the remaining 275 data points more clearly.
(TIF)

S3 Fig Fig 6D with all outliers visible. The same five simulations which resulted in fully arrested plaques in S2 Fig also result in dramatically lower average extracellular virus AUC. These outliers were cropped out in the original figure to show the distribution of the remaining 275 data points more clearly.
(TIF)

S4 Fig Simulated IFN_e_ exposure before infection protects cells from plaque formation. The plate was washed with extracellular Type-I interferons (Simulation initial conditions in Main Text Table 3), then a single cell was infected at the center of the plate. Since cell health is the median of all live cells’ health, the initially infected cell dying ~16 hours caused a brief increase in median cell health. No interferon-triggered death mechanism or resource limitations are present, leading to boundless amplification of the cytokine signal after the virus has been cleared. Bold lines are median of 20 replicas; shaded areas represent the 5^th^ and 95^th^ percentiles.
(TIF)

S5 Fig Local sensitivity analysis of baseline simulation. Down and up columns give average value change for each of the metrics when the parameter is varied -25% (down) and +25% (up) of their baseline value. These changes are shaded red for positive changes and blue for negative changes in the metric, with intensity normalized to the largest change within both columns of each metric. p-values are the statistical significance of the change, given the standard deviation of the stochastic simulations over 20 replicas (See S1 Fig). p-values < 0.01 are highlighted in yellow. Note that τ_IRF7_ has a large response in baseline IFN_e_ Max because the baseline value for τ_IRF7_ lies near the stability criterion of *τ*_*IRF7*_ > 0.75, so the 25% decrease leads to a numerically unstable system.
(TIF)

S6 Fig Local sensitivity analysis with elevated paracrine signaling. This case corresponds to a 15x increase in the phosphorylation rate of STATP via the JAK/STAT pathway (Main Text Table 2, parameter *k*_*STATP*,*IFNe*._ Value changed from baseline of 45.922 μM hr^-1^ to 688.83 μM hr^-1^). Down and up columns give average value change for each of the metrics when the parameter is varied -25% (down) and +25% (up) of their baseline value. These changes are shaded red for positive changes and blue for negative changes in the metric, with intensity normalized to the largest change within both columns of each metric. p-values are the statistical significance of the change, given the standard deviation of the stochastic simulations over 20 replicas (See S1 Fig). p-values < 0.01 are highlighted in yellow.
(TIF)

S7 Fig Local sensitivity with an elevated interferon diffusion coefficient (15x baseline or 540 μm^2^ s^-1^). Plaques are arrested by the paracrine signal diffusion significantly faster than viral spread. Down and up columns give average value change for each of the metrics when the parameter is varied -25% (down) and +25% (up) of their baseline value. These changes are shaded red for positive changes and blue for negative changes in the metric, with intensity normalized to the largest change within both columns of each metric. p-values are the statistical significance of the change, given the standard deviation of the stochastic simulations over 20 replicas (See S1 Fig). p-values < 0.01 are highlighted in yellow.
(TIF)

S8 Fig The effect of changing the of viral replication rate (*k*_*V*,*V*_) on plaque diameter. A. Plaque growth over 80 hours post-infection. B. Tracking of cell types (uninfected, U, eclipse infected, I1, virus releasing, I2, and dead, D) for plaque growth dynamics corresponding to plaques in A. Center lines represent median over 20 replicas; shaded areas are the 5^th^ and 95^th^ percentiles. (C) Viral replication rate, *k*_*V*,*V*_, multipliers versus growth rate of a single plaque at the end of the simulation at 80 hours and (D) the area under the curve (AUC) for both average extracellular virus and (E) average extracellular interferon (full data with 4 additional outliers available in S11 Fig) on log scales. Lowering viral replication below the nonspecific viral clearance rate prevents plaque formation. Higher replications have exponential changes in system metrics. Lowering viral replication speed slows, and can even prevent plaque growth, as virus is cleared from the extracellular environment more quickly relative to the rate of production and release. This limits the size of plaques *in vitro* and lesion size *in vivo*. Higher viral replication leads to exponentially faster growth and larger lesions since virus replication is self-amplifying. Viral titer growth follows an exponential growth curve; however, the radial growth of the plaques is linear. These replicate biological observations.
(TIF)

S9 Fig A parameter sweep of *β* (rate of transition from uninfected (U) to eclipse phase (I1) cells) from 0.01x to 100x baseline reveals steady growth increases. Plaques still form at any nonzero value. A. Plaque growth over 80 hours post-infection. B. Tracking of cell types (U, I1, virus releasing, I2, and dead, D) for plaque growth dynamics corresponding to plaques in A. Center lines represent median over 20 replicas; shaded areas are the 5^th^ and 95^th^ percentiles. (C) *β* parameter multipliers versus growth rate of a single plaque at the end of the simulation at 80 hours and (D) the area under the curve (AUC) for both average extracellular virus and (E) average extracellular IFN on log scales. Full data with 14 additional outliers for average extracellular interferon and 1 additional outlier for the average extracellular virus are available in S12 and S13 Figs, respectively. Higher virus infectivity resulted in higher proportions of dead cells within the plaque. Note a non-monotonic trend; natural virus infectivity leads to a minimum production of IFN_e_. Decreases and increases in *β* both led to higher IFN_e_ production. Viruses have differing encapsulation proteins, genome sizes, and relative production of nonstructural proteins while replicating within a host cell. These differences lead to variable virus replication rates, represented in the model by *k*_*V*,*V*_.
(TIF)

S10 Fig Elevated IFN diffusion coefficient (*D_IFNe_*) leads to plaque arrest. A. Plaque growth over 80 hours post-infection. B. Tracking of cell types (uninfected, U, eclipse infected, I1, virus releasing, I2, and dead, D) for plaque growth dynamics corresponding to plaques in A. Center lines represent median over 20 replicas; shaded areas are the 5^th^ and 95^th^ percentiles. (C) Extracellular interferon diffusion, *D_IFNe_*, parameter multipliers versus growth rate of a single plaque at the end of the simulation at 80 hours and (D) the area under the curve (AUC) for both average extracellular virus (full data with 1 additional outlier available in S14 Fig) and (E) average extracellular interferon on log scales. Plaque growth loses linearity and is arrested after 10x increases.
(TIF)

S11 Fig S8E Fig with all outliers visible. 4 outlier simulations resulted in much higher average extracellular interferon AUC. These outliers were cropped out in the original figure to show the distribution of the remaining 276 data points more clearly.
(TIF)

S12 Fig S9E Fig with all outliers visible. 14 outlier simulations resulted in significantly higher average extracellular interferon AUC. These outliers were cropped out in the original figure to show the distribution of the remaining 266 data points more clearly.
(TIF)

S13 Fig S9D Fig with all outliers visible. A single outlier simulation resulted in a much lower average extracellular virus AUC. This outlier was cropped out in the original figure to show the distribution of the remaining 279 data points more clearly.
(TIF)

S14 Fig S10D Fig with all outliers visible. A single outlier simulation resulted in a much lower average extracellular virus AUC. This outlier was cropped out in the original figure to show the distribution of the remaining 279 data points more clearly.
(TIF)
