# Supplementary figures and images for: Multicellular spatial model of RNA virus replication and interferon responses reveals factors controlling plaque growth dynamics
Source: PLoS Comput Biol. 2021 Oct 25;17(10):e1008874. doi: 10.1371/journal.pcbi.1008874 (PMC8608315; doi:10.1371/journal.pcbi.1008874)

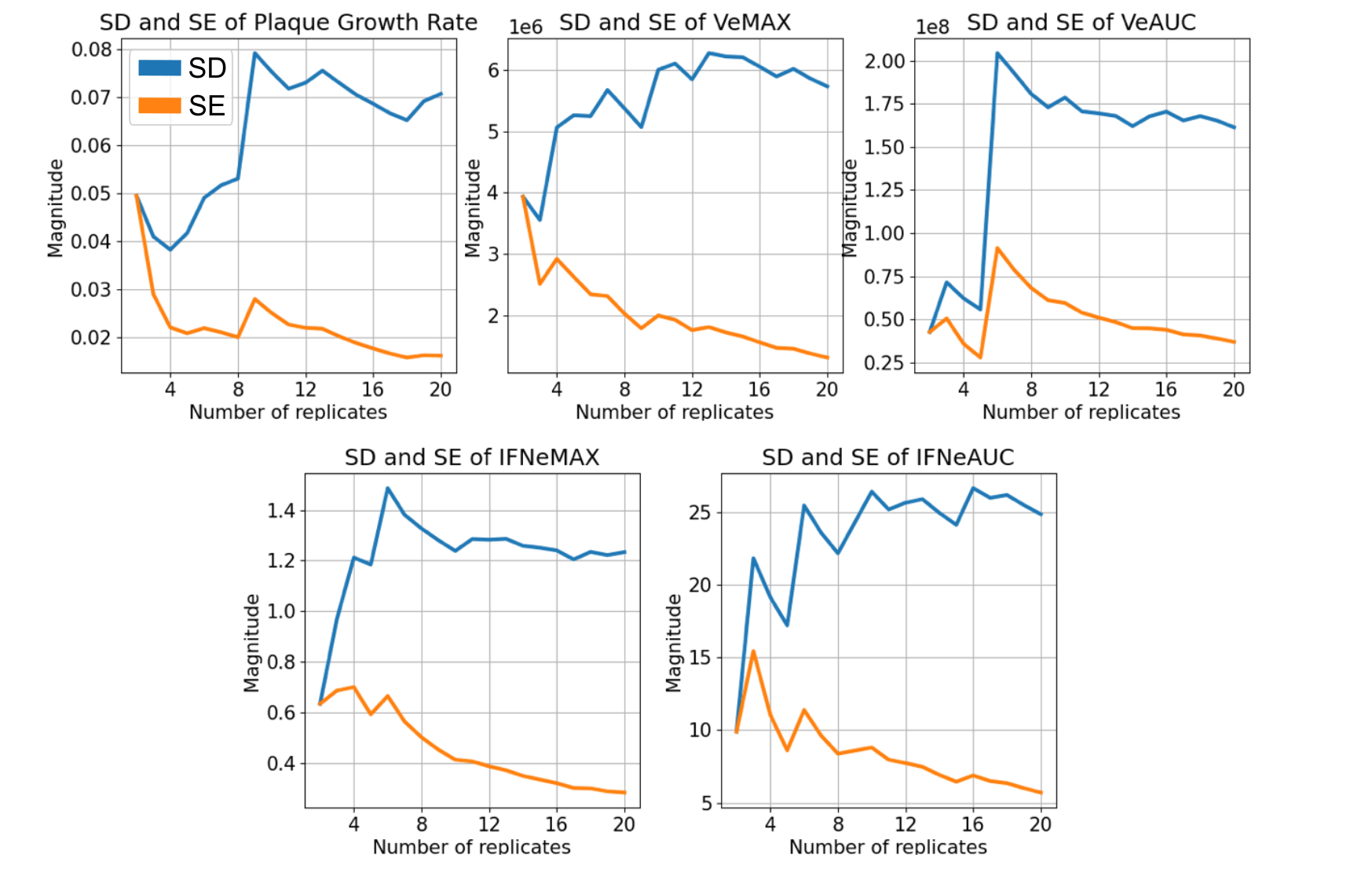

Supplement: S1 Fig — Used to justify n = 20 replicas for sensitivity analyses and parameter sweeps. Standard deviation (SD) is blue while standard error (SE) is orange. (TIF) [file pcbi.1008874.s001.tif]

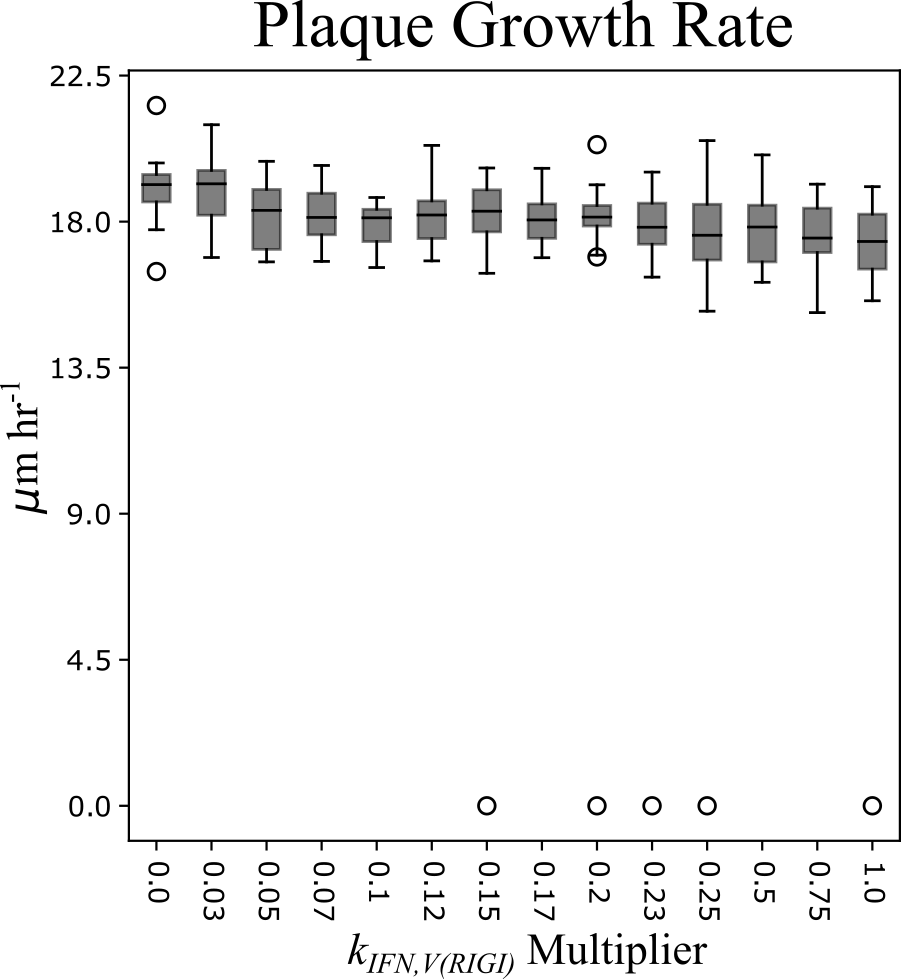

Supplement: S2 Fig — Five outlier simulations of increased kIFN,V(RIGI) activity over baseline resulted in fully arrested plaques by 80 hours post-infection. These outliers were cropped out in the original figure to show the distribution of the remaining 275 data points more clearly. (TIF) [file pcbi.1008874.s002.tif]

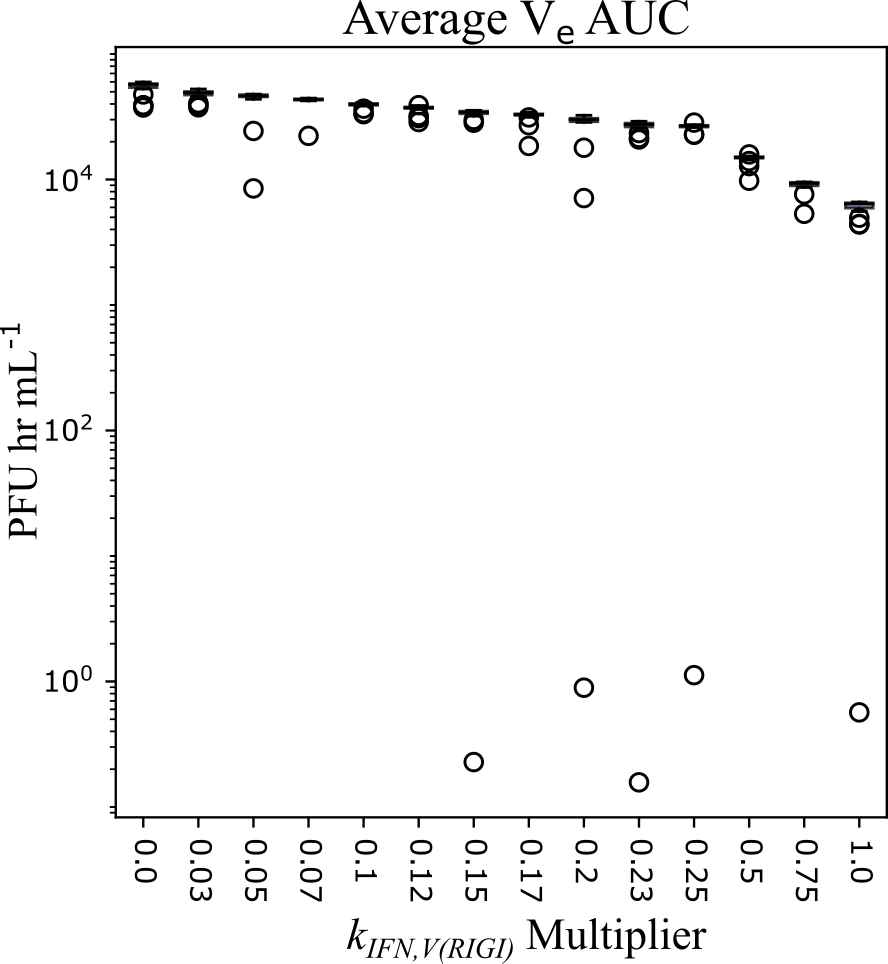

Supplement: S3 Fig — The same five simulations which resulted in fully arrested plaques in S2 Fig also result in dramatically lower average extracellular virus AUC. These outliers were cropped out in the original figure to show the distribution of the remaining 275 data points more clearly. (TIF) [file pcbi.1008874.s003.tif]

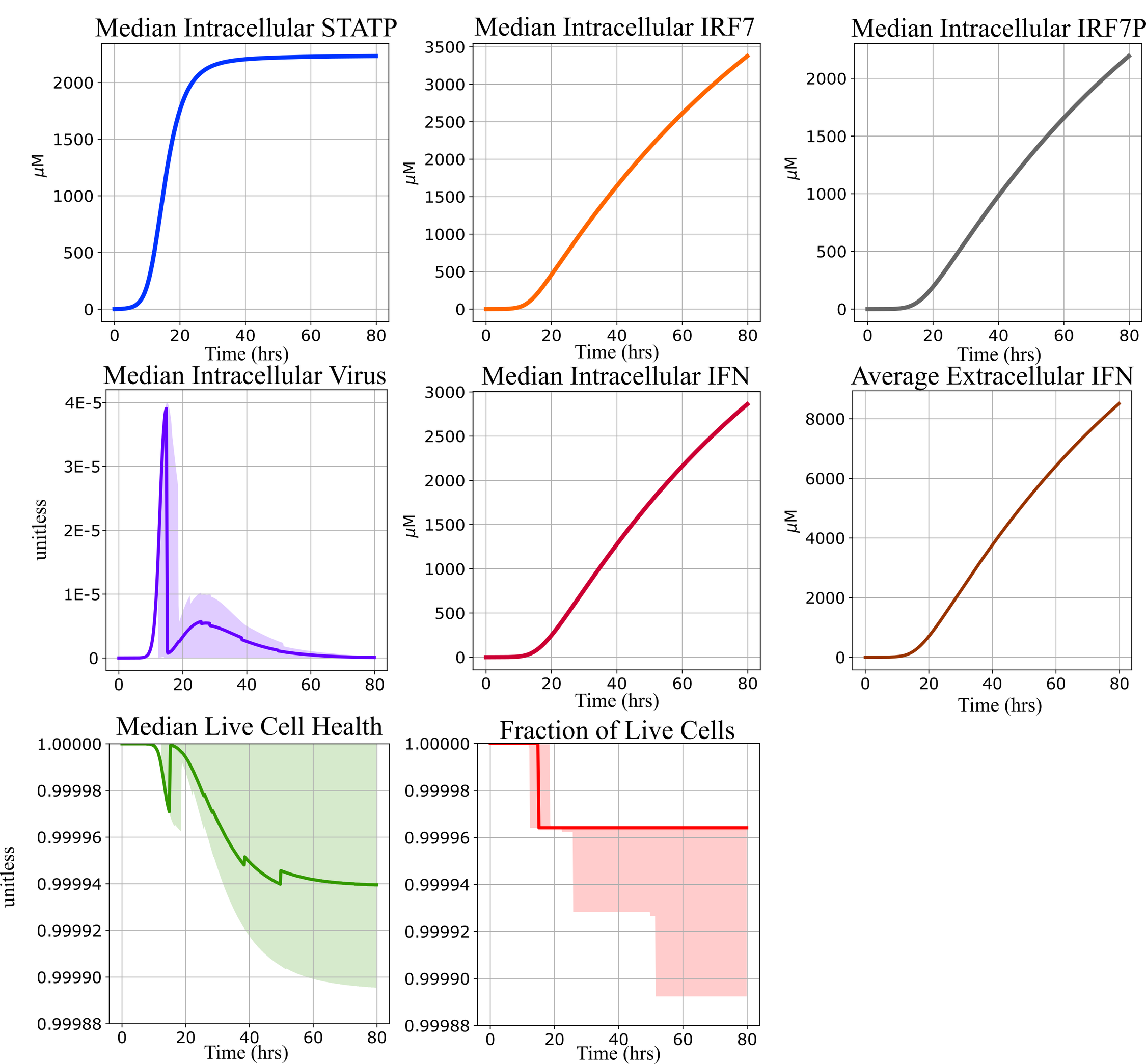

Supplement: S4 Fig — The plate was washed with extracellular Type-I interferons (Simulation initial conditions in Main Text Table 3), then a single cell was infected at the center of the plate. Since cell health is the median of all live cells’ health, the initially infected cell dying ~16 hours caused a brief increase in median cell health. No interferon-triggered death mechanism or resource limitations are present, leading to boundless amplification of the cytokine signal after the virus has been cleared. Bold lines are median of 20 replicas; shaded areas represent the 5th and 95th percentiles. (TIF) [file pcbi.1008874.s004.tif]

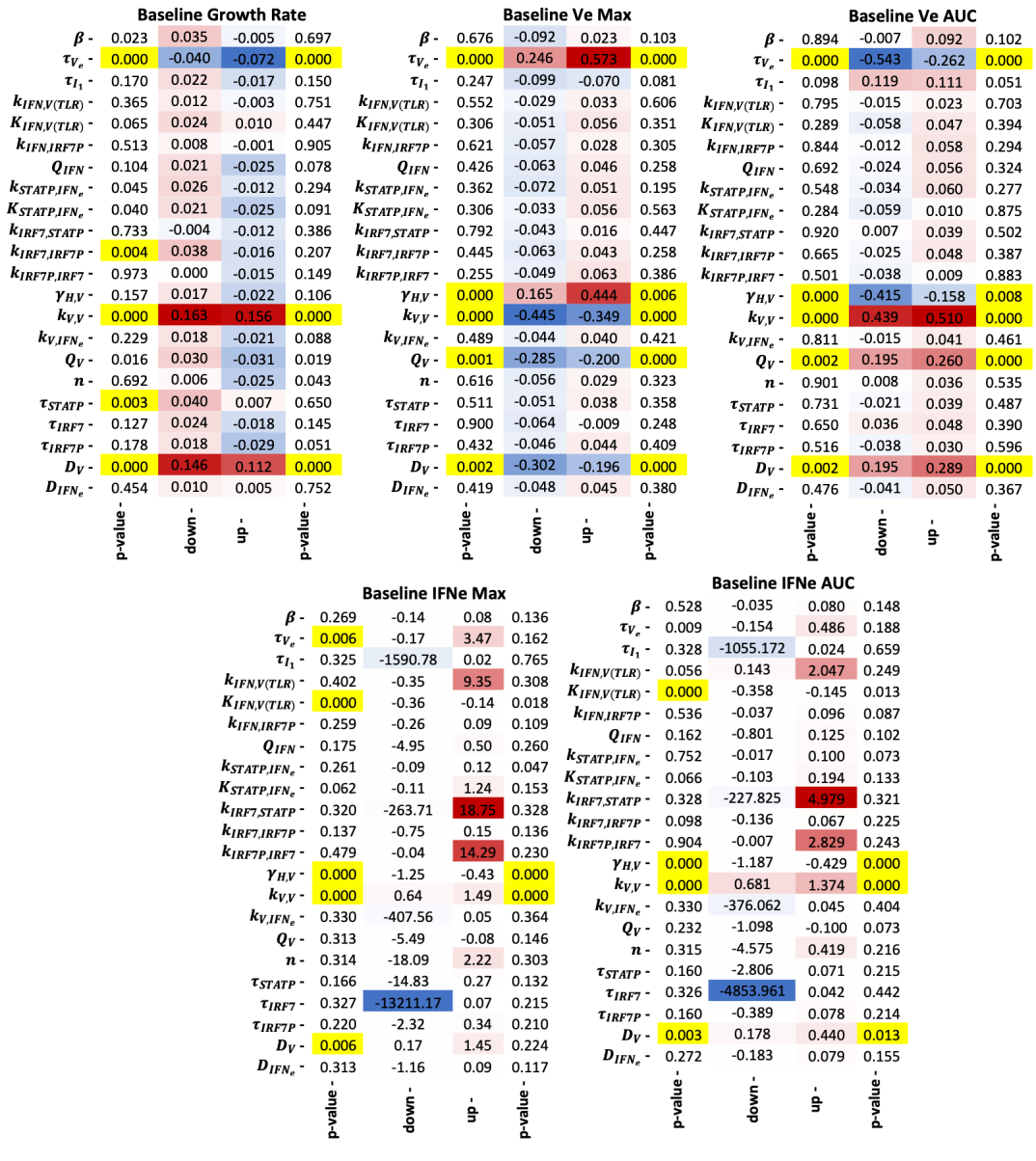

Supplement: S5 Fig — Down and up columns give average value change for each of the metrics when the parameter is varied -25% (down) and +25% (up) of their baseline value. These changes are shaded red for positive changes and blue for negative changes in the metric, with intensity normalized to the largest change within both columns of each metric. p-values are the statistical significance of the change, given the standard deviation of the stochastic simulations over 20 replicas (See S1 Fig). p-values < 0.01 are highlighted in yellow. Note that τIRF7 has a large response in baseline IFNe Max because the baseline value for τIRF7 lies near the stability criterion of τIRF7 > 0.75, so the 25% decrease leads to a numerically unstable system. (TIF) [file pcbi.1008874.s005.tif]

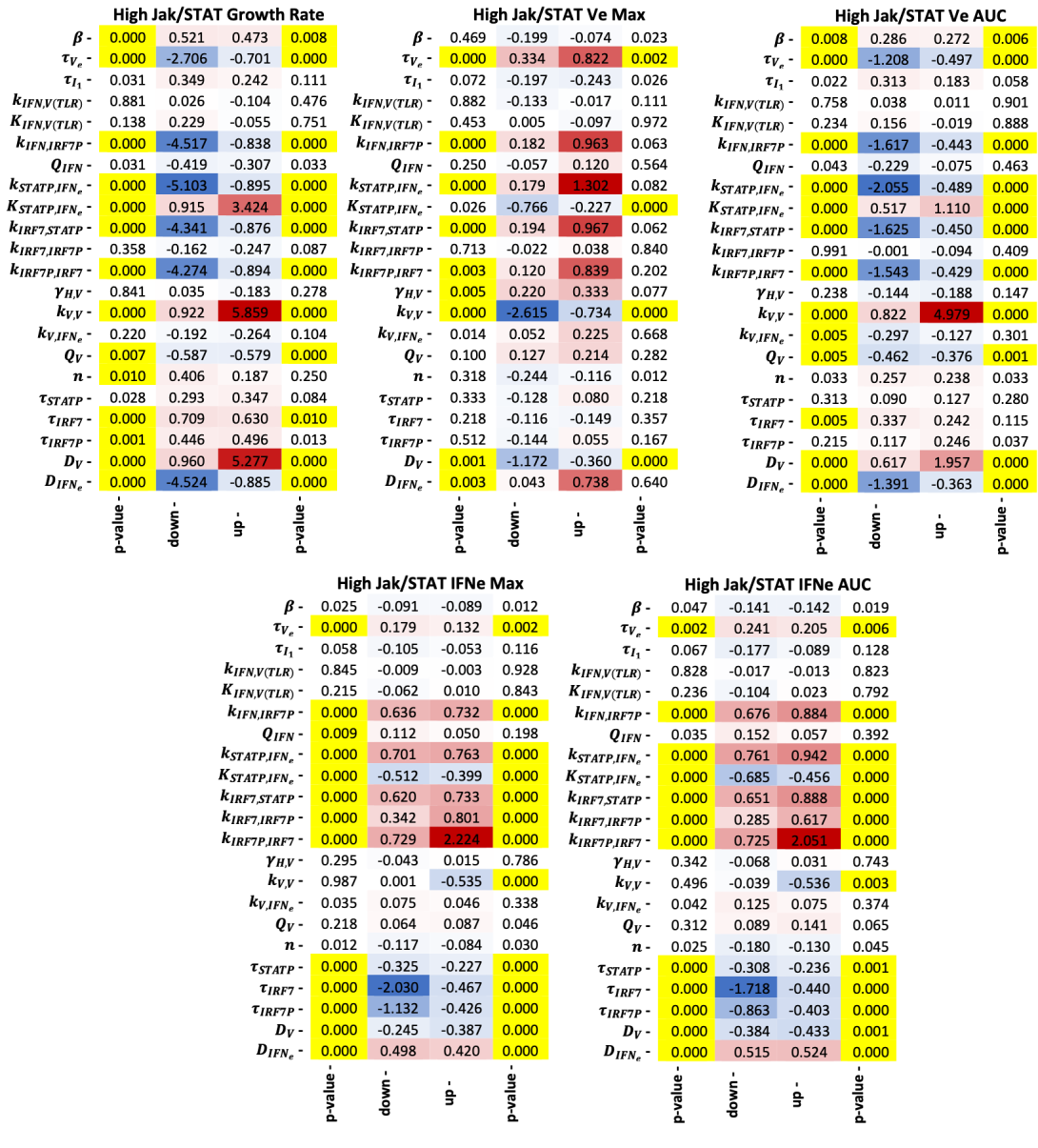

Supplement: S6 Fig — This case corresponds to a 15x increase in the phosphorylation rate of STATP via the JAK/STAT pathway (Main Text Table 2, parameter kSTATP,IFNe. Value changed from baseline of 45.922 μM hr-1 to 688.83 μM hr-1). Down and up columns give average value change for each of the metrics when the parameter is varied -25% (down) and +25% (up) of their baseline value. These changes are shaded red for positive changes and blue for negative changes in the metric, with intensity normalized to the largest change within both columns of each metric. p-values are the statistical significance of the change, given the standard deviation of the stochastic simulations over 20 replicas (See S1 Fig). p-values < 0.01 are highlighted in yellow. (TIF) [file pcbi.1008874.s006.tif]

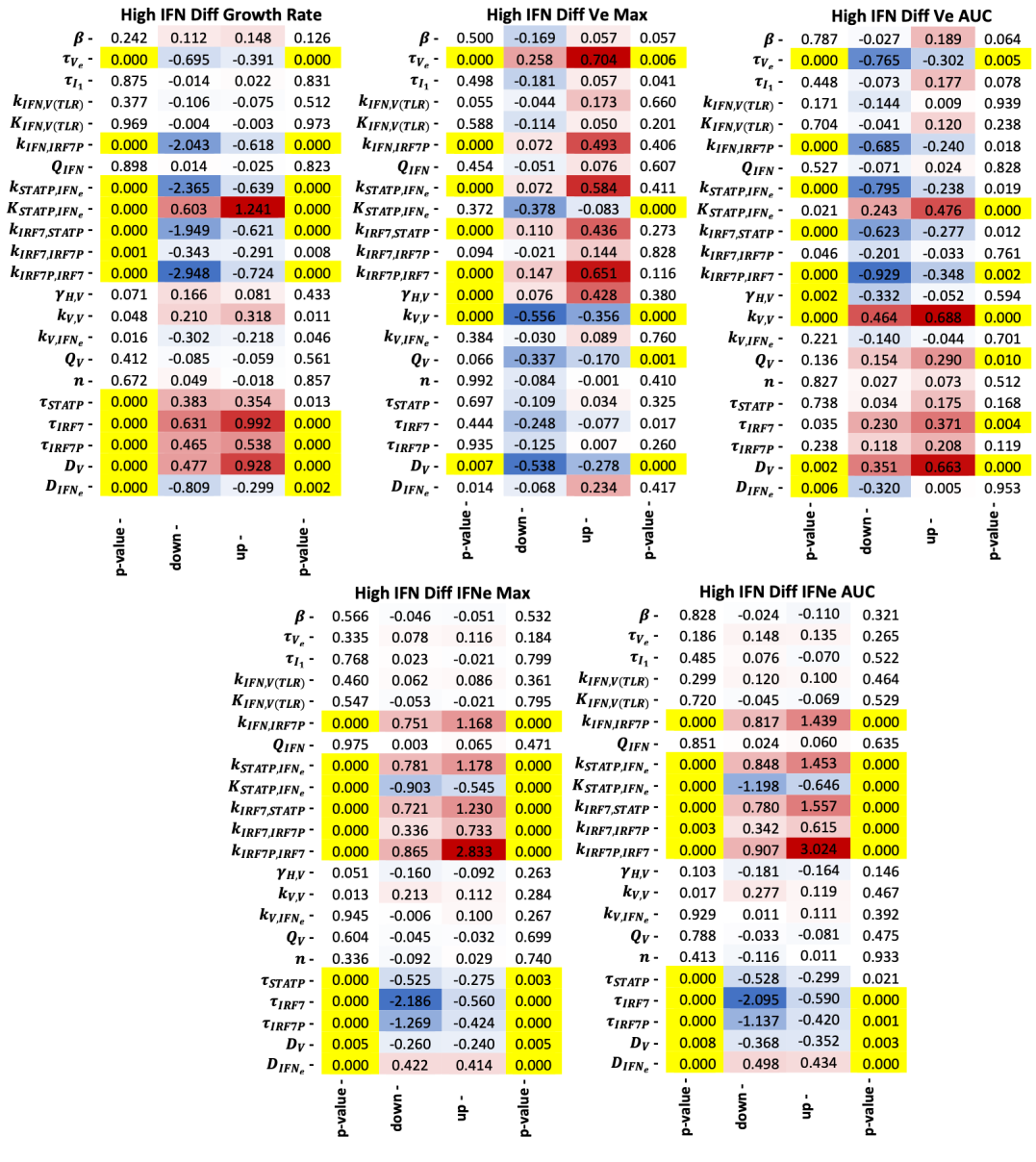

Supplement: S7 Fig — Plaques are arrested by the paracrine signal diffusion significantly faster than viral spread. Down and up columns give average value change for each of the metrics when the parameter is varied -25% (down) and +25% (up) of their baseline value. These changes are shaded red for positive changes and blue for negative changes in the metric, with intensity normalized to the largest change within both columns of each metric. p-values are the statistical significance of the change, given the standard deviation of the stochastic simulations over 20 replicas (See S1 Fig). p-values < 0.01 are highlighted in yellow. (TIF) [file pcbi.1008874.s007.tif]

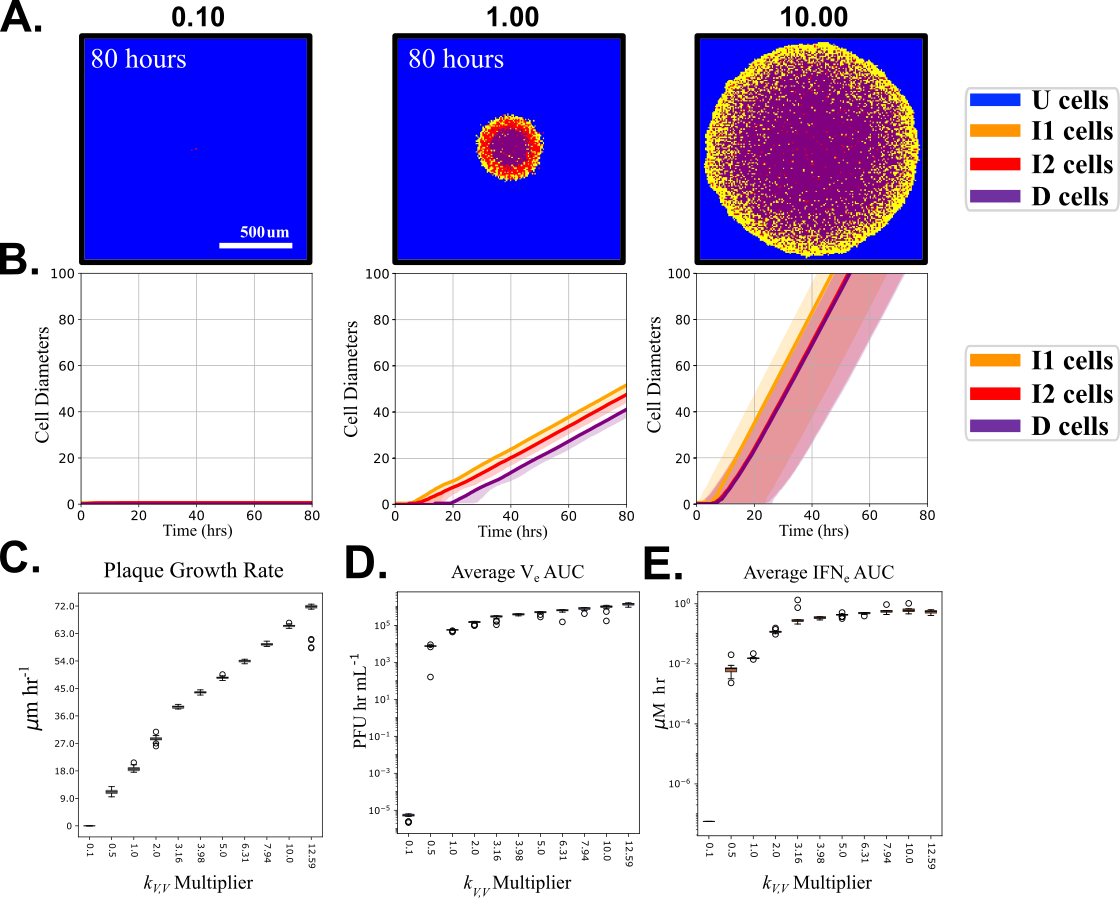

Supplement: S8 Fig — A. Plaque growth over 80 hours post-infection. B. Tracking of cell types (uninfected, U, eclipse infected, I1, virus releasing, I2, and dead, D) for plaque growth dynamics corresponding to plaques in A. Center lines represent median over 20 replicas; shaded areas are the 5th and 95th percentiles. (C) Viral replication rate, kV,V, multipliers versus growth rate of a single plaque at the end of the simulation at 80 hours and (D) the area under the curve (AUC) for both average extracellular virus and (E) average extracellular interferon (full data with 4 additional outliers available in S11 Fig) on log scales. Lowering viral replication below the nonspecific viral clearance rate prevents plaque formation. Higher replications have exponential changes in system metrics. Lowering viral replication speed slows, and can even prevent plaque growth, as virus is cleared from the extracellular environment more quickly relative to the rate of production and release. This limits the size of plaques in vitro and lesion size in vivo. Higher viral replication leads to exponentially faster growth and larger lesions since virus replication is self-amplifying. Viral titer growth follows an exponential growth curve; however, the radial growth of the plaques is linear. These replicate biological observations. (TIF) [file pcbi.1008874.s008.tif]

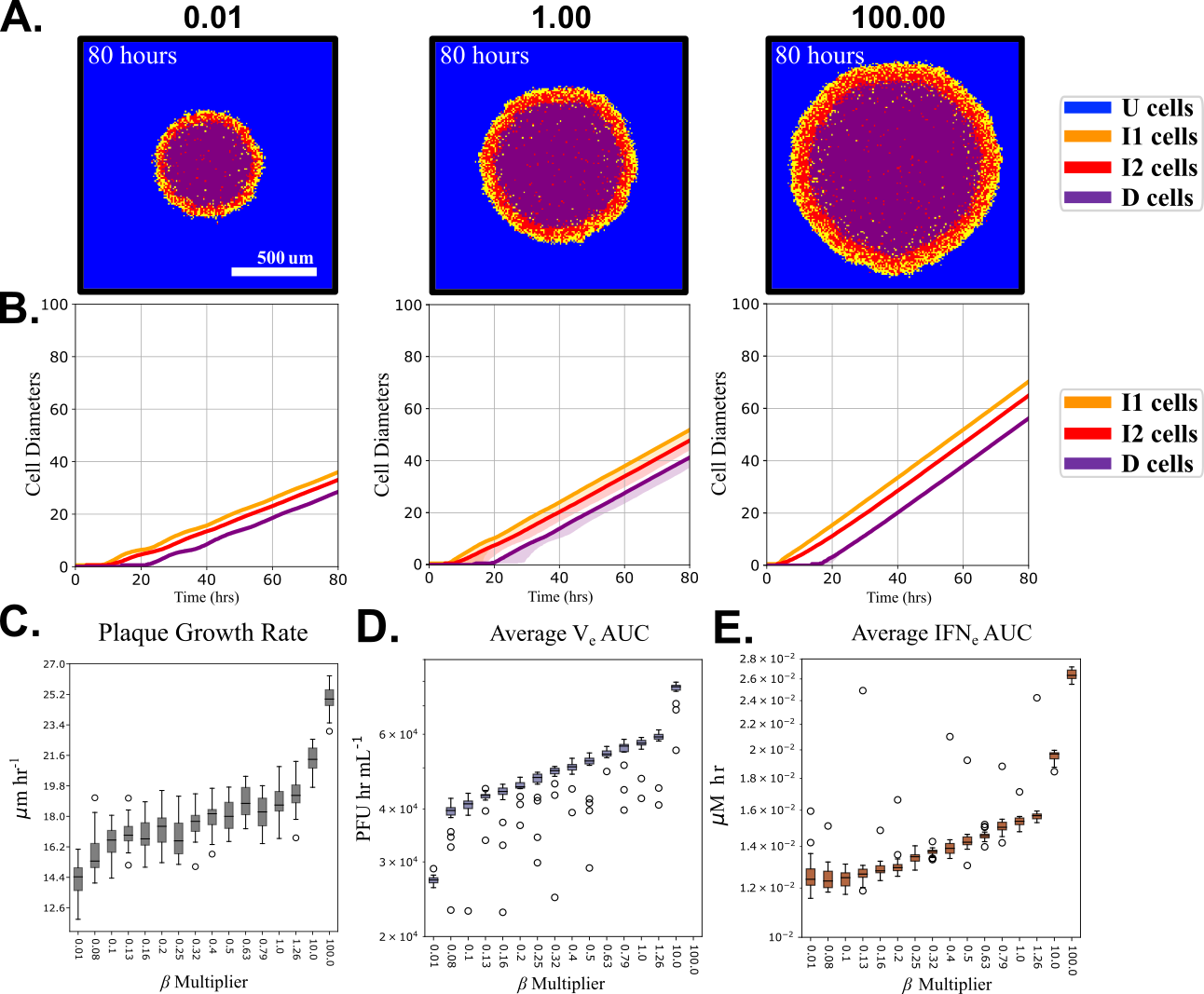

Supplement: S9 Fig — Plaques still form at any nonzero value. A. Plaque growth over 80 hours post-infection. B. Tracking of cell types (U, I1, virus releasing, I2, and dead, D) for plaque growth dynamics corresponding to plaques in A. Center lines represent median over 20 replicas; shaded areas are the 5th and 95th percentiles. (C) β parameter multipliers versus growth rate of a single plaque at the end of the simulation at 80 hours and (D) the area under the curve (AUC) for both average extracellular virus and (E) average extracellular IFN on log scales. Full data with 14 additional outliers for average extracellular interferon and 1 additional outlier for the average extracellular virus are available in S12 and S13 Figs, respectively. Higher virus infectivity resulted in higher proportions of dead cells within the plaque. Note a non-monotonic trend; natural virus infectivity leads to a minimum production of IFNe. Decreases and increases in β both led to higher IFNe production. Viruses have differing encapsulation proteins, genome sizes, and relative production of nonstructural proteins while replicating within a host cell. These differences lead to variable virus replication rates, represented in the model by kV,V. (TIF) [file pcbi.1008874.s009.tif]

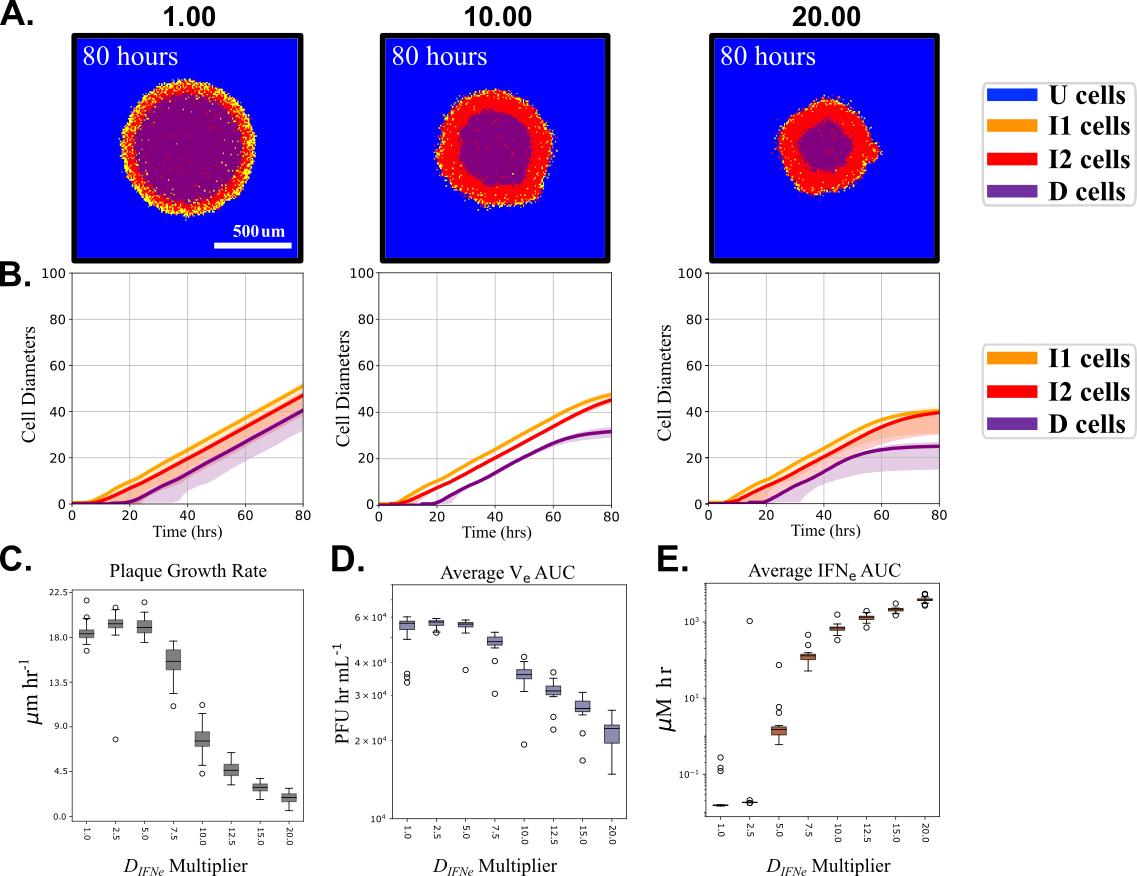

Supplement: S10 Fig — A. Plaque growth over 80 hours post-infection. B. Tracking of cell types (uninfected, U, eclipse infected, I1, virus releasing, I2, and dead, D) for plaque growth dynamics corresponding to plaques in A. Center lines represent median over 20 replicas; shaded areas are the 5th and 95th percentiles. (C) Extracellular interferon diffusion, DIFNe, parameter multipliers versus growth rate of a single plaque at the end of the simulation at 80 hours and (D) the area under the curve (AUC) for both average extracellular virus (full data with 1 additional outlier available in S14 Fig) and (E) average extracellular interferon on log scales. Plaque growth loses linearity and is arrested after 10x increases. (TIF) [file pcbi.1008874.s010.tif]

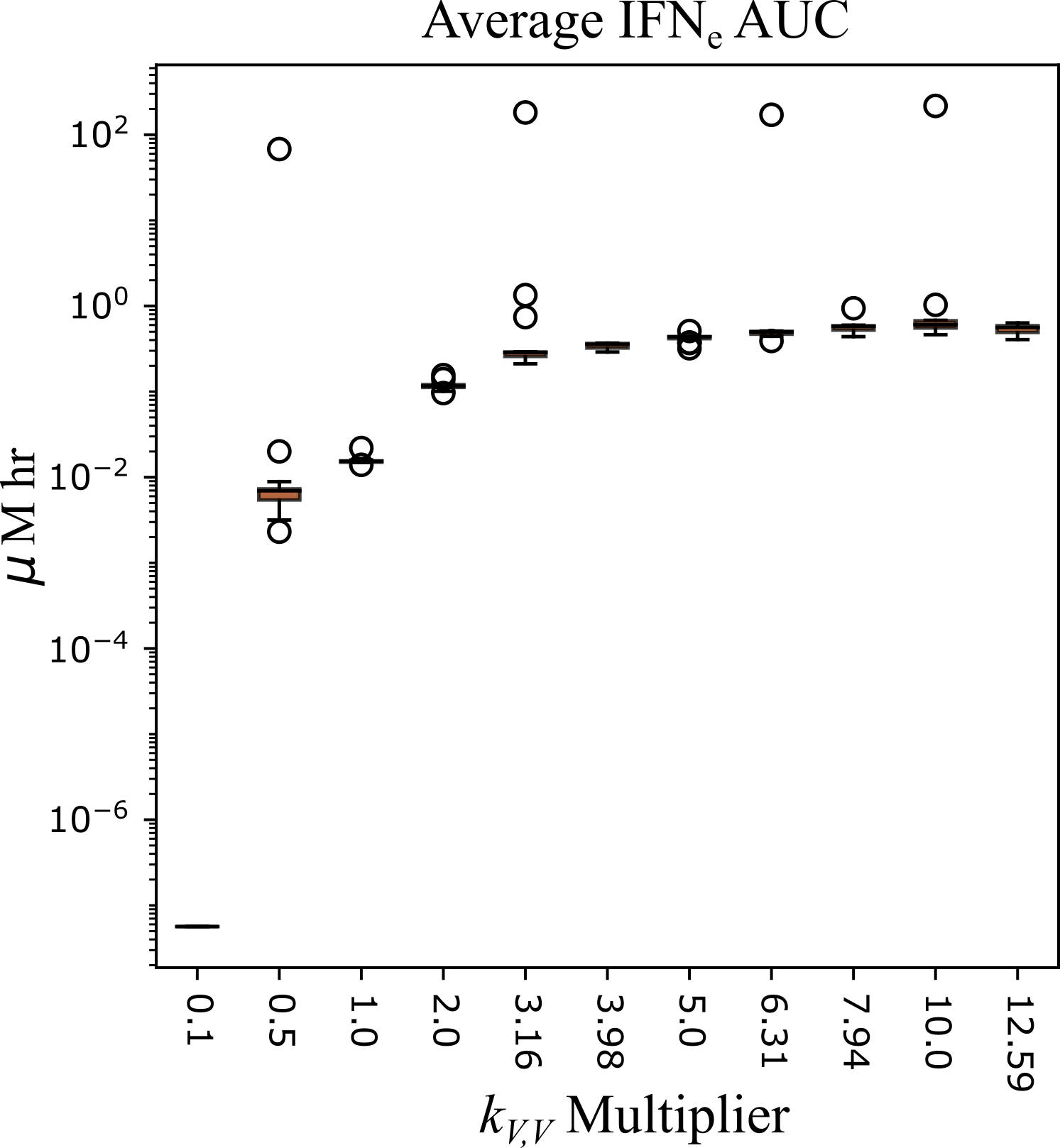

Supplement: S11 Fig — 4 outlier simulations resulted in much higher average extracellular interferon AUC. These outliers were cropped out in the original figure to show the distribution of the remaining 276 data points more clearly. (TIF) [file pcbi.1008874.s011.tif]

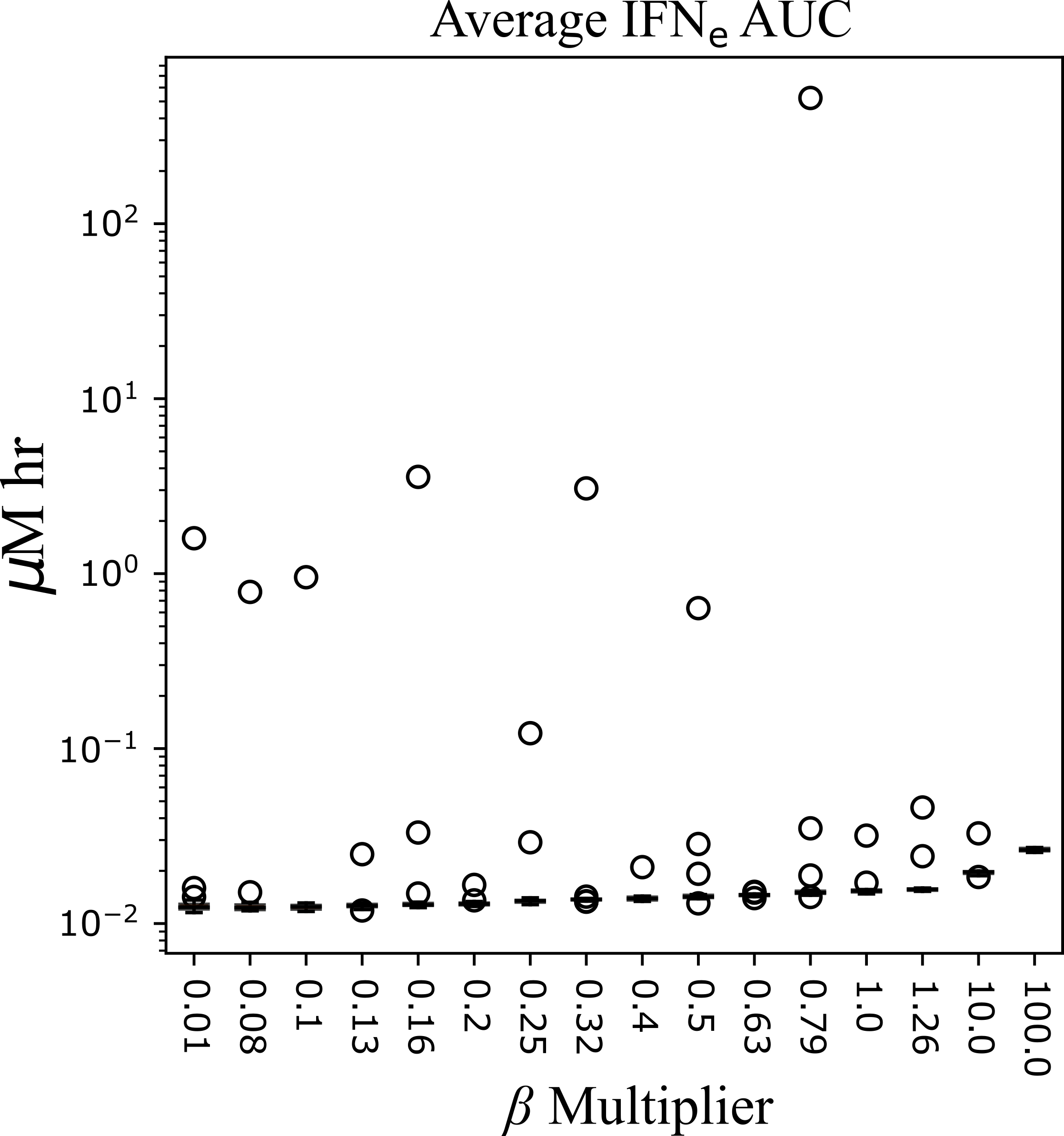

Supplement: S12 Fig — 14 outlier simulations resulted in significantly higher average extracellular interferon AUC. These outliers were cropped out in the original figure to show the distribution of the remaining 266 data points more clearly. (TIF) [file pcbi.1008874.s012.tif]

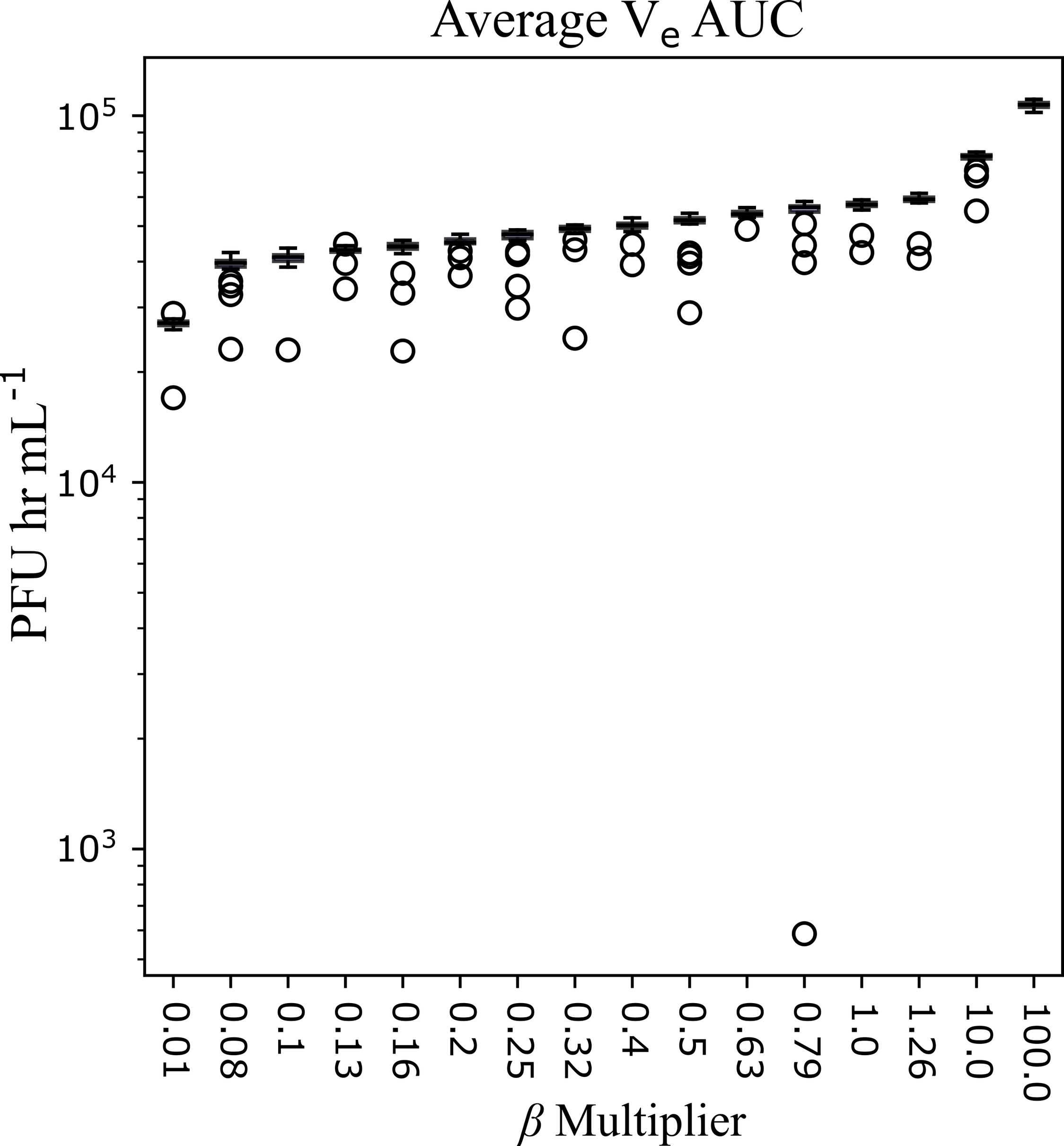

Supplement: S13 Fig — A single outlier simulation resulted in a much lower average extracellular virus AUC. This outlier was cropped out in the original figure to show the distribution of the remaining 279 data points more clearly. (TIF) [file pcbi.1008874.s013.tif]

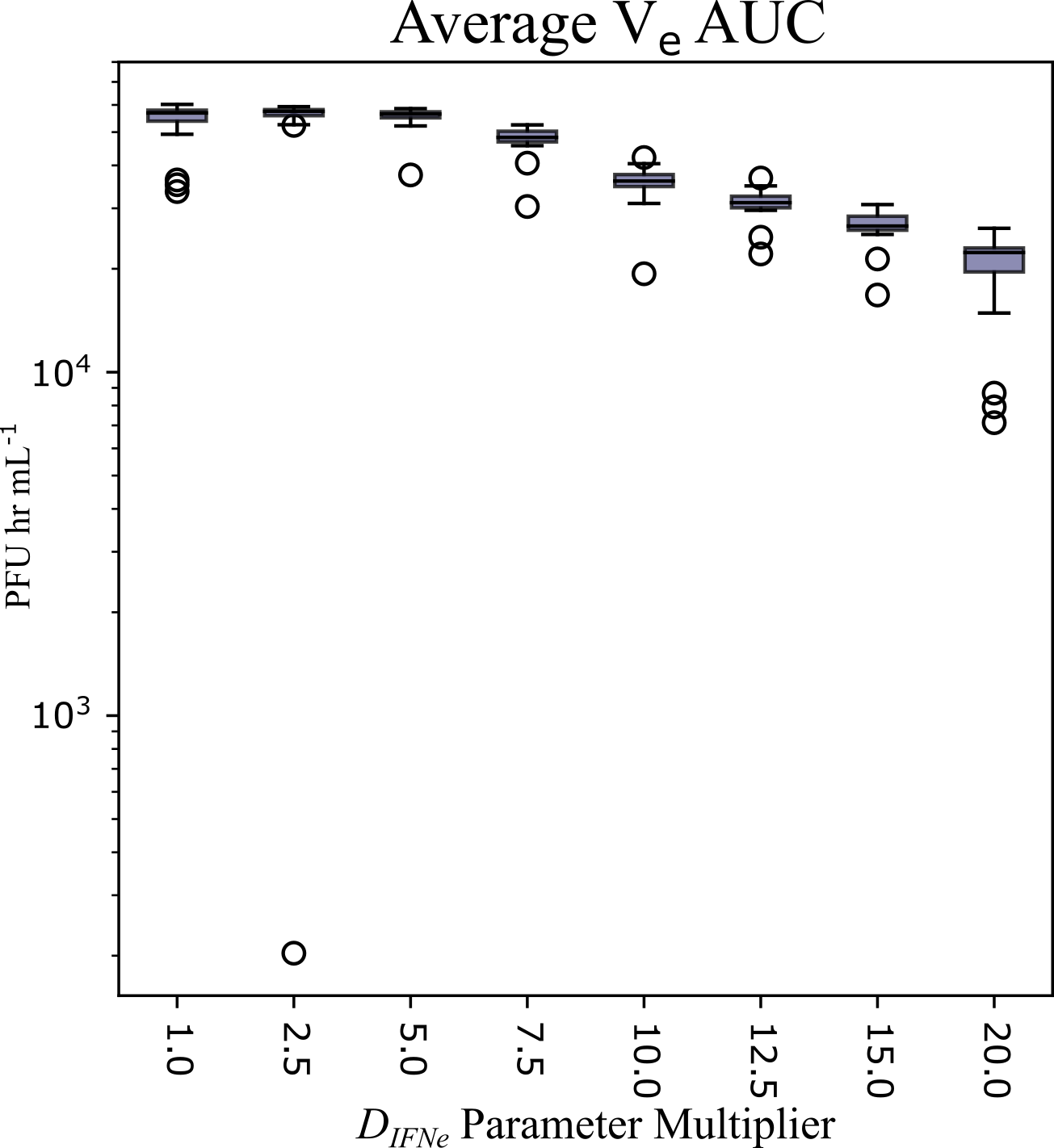

Supplement: S14 Fig — A single outlier simulation resulted in a much lower average extracellular virus AUC. This outlier was cropped out in the original figure to show the distribution of the remaining 279 data points more clearly. (TIF) [file pcbi.1008874.s014.tif]
